# Persistent luminescent nanophosphors for applications in cancer theranostics, biomedical, imaging and security

**DOI:** 10.1016/j.mtbio.2023.100860

**Published:** 2023-11-19

**Authors:** Umer Mushtaq, Irfan Ayoub, Vijay Kumar, Vishal Sharma, Hendrik C. Swart, Elham Chamanehpour, Horst-Günter Rubahn, Yogendra Kumar Mishra

**Affiliations:** a Department of Physics, National Institute of Technology Srinagar, Jammu and Kashmir, 190006, India; b Institute of Forensic Science & Criminology, Panjab University, Chandigarh, 160014, India; c Department of Physics, University of the Free State, P.O. Box 339, Bloemfontein, ZA9300, South Africa; d NanoSYD, Mads Clausen Institute, University of Southern Denmark, Alsion 2, Sønderborg, 6400, Denmark

**Keywords:** Persistent luminescence, Nanophosphors, Cancer theranostics, Biomedical, Imaging, Security technologies, Anti-counterfeiting, Latent fingerprinting

## Abstract

The extraordinary and unique properties of persistent luminescent (PerLum) nanostructures like storage of charge carriers, extended afterglow, and some other fascinating characteristics like no need for in-situ excitation, and rechargeable luminescence make such materials a primary candidate in the fields of bio-imaging and therapeutics. Apart from this, due to their extraordinary properties they have also found their place in the fields of anti-counterfeiting, latent fingerprinting (LPF), luminescent markings, photocatalysis, solid-state lighting devices, glow-in-dark toys, *etc.* Over the past few years, persistent luminescent nanoparticles (PLNPs) have been extensively used for targeted drug delivery, bio-imaging guided photodynamic and photo-thermal therapy, biosensing for cancer detection and subsequent treatment, latent fingerprinting, and anti-counterfeiting owing to their enhanced charge storage ability, in-vitro excitation, increased duration of time between excitation and emission, low tissue absorption, high signal-to-noise ratio, etc. In this review, we have focused on most of the key aspects related to PLNPs, including the different mechanisms leading to such phenomena, key fabrication techniques, properties of hosts and different activators, emission, and excitation characteristics, and important properties of trap states. This review article focuses on recent advances in cancer theranostics with the help of PLNPs. Recent advances in using PLNPs for anti-counterfeiting and latent fingerprinting are also discussed in this review.

## Graphical abstract

**Figure:**
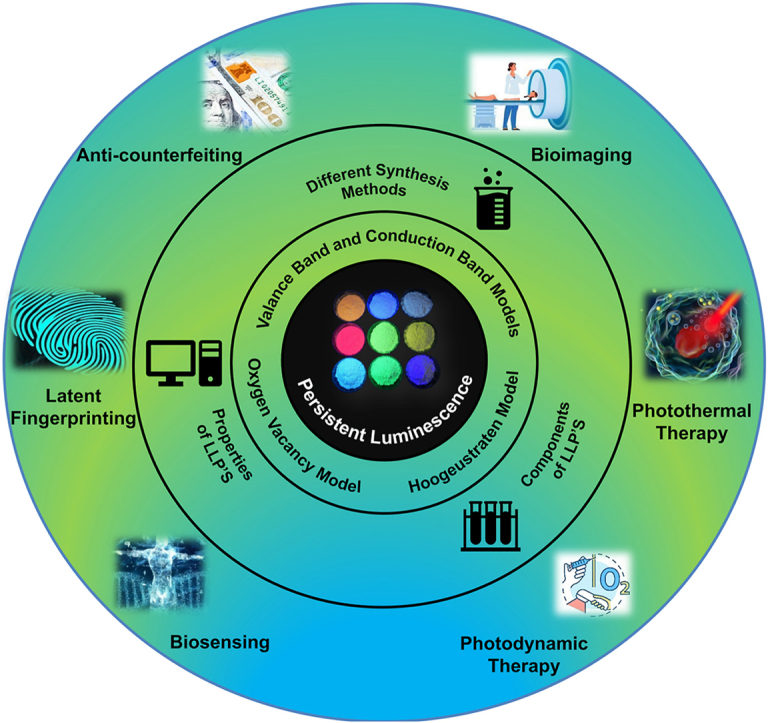

## 1 Introduction

**Fig. 1:**
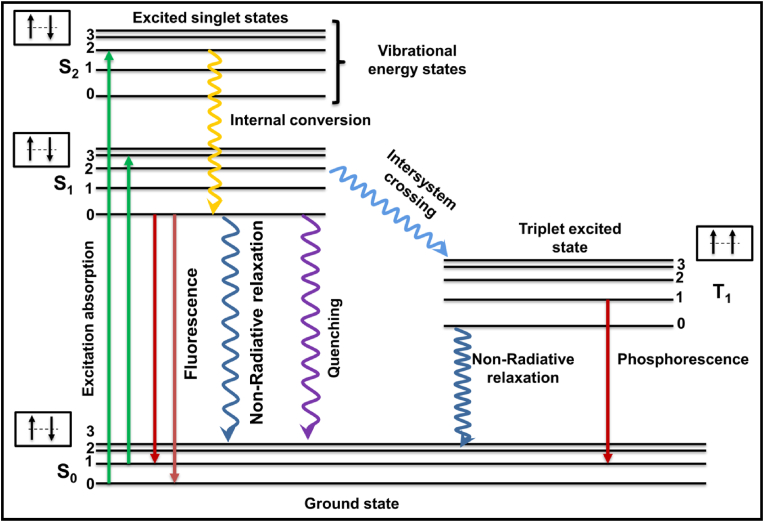
Jablonski diagram explaining fluorescence (S_1_–S_0_ transition) and phosphorescence (T_1_-S_0_ transition) along with other undergoing transitions.

Luminescence is a term commonly used to describe the excitation of electrons using UV or visible radiation, whose energy is then emitted as visible light. The excitation source can be of a chemical, biological, or physical nature [1]. In this review, our focus is on radiation sources of a physical nature, for which the term photoluminescence would be used [2]. It is a process in which a molecule is excited using visible or ultraviolet photons, sending its electrons to excited electronic states. Photoluminescence can be categorized into two categories: one is fluorescence, which is a transition from a singlet excited state to a singlet ground state, thus being spin-allowed and hence causing instantaneous photon emission with a lifetime of the order of nanoseconds after excitation [3,4]. The second is phosphorescence, which is the phenomenon of delayed photon emission post-excitation caused by the transition from an excited triplet state to a singlet ground state; as this transition is not spin-mediated (mediated by spin-orbit coupling), de-excitation takes a longer duration, ranging from a few milliseconds to some minutes [5]. With phosphorescence, the emission intensity shows a gradual decrease over time, whereas with fluorescence, the emission intensity shows an exponential decrease over time. After the initial absorption of visible or UV light, the molecule goes into an excited electronic state, and then, after being subjected to collisions with surrounding molecules, it loses energy non-radiatively, thus going down to the lowest vibrational level of the excited molecular state [3]. Finally, the molecule undergoes a radiative transition from the lowest vibrational level of the excited molecular state to the ground state. Fluorescence is a singlet-singlet transition that follows Hund's rule and thus has a very short afterglow of the order of nanoseconds (S_1_–S_0_), but the long-lasting afterglow (phosphorescence) is caused by the spin-forbidden triplet-singlet (T_1_-S_0_) transition delayed because of intersystem crossing [6]. These transitions can be clearly understood using the Jablonski diagram shown in Fig. 1 [7]. Trapping of charge carriers in trap states leads to extending the duration of the afterglow in the case of phosphorescence to a couple of hours, preferably known as persistent luminescence (PerLum) [8]. This phenomenon can be understood as the continuous emission of radiation for extended durations after the removal of the excitation source in some luminescent materials called PerLum materials and sometimes afterglow [9,10]. In 1996, Matsuzawa et al. [8], reported a green-emitting SrAl_2_O_4_: Eu^2+^, Dy^3+^ phosphor with very bright and long-lasting phosphorescence that was over 10 times brighter than ZnS: Cu, Co. This aluminate phosphor exhibited properties of extreme brightness and an extended afterglow lasting for over 30 h before the emission intensity was observed to drop to 0.32 mcd/m^2^, which is the minimum emission intensity detectable by the naked human eye [8]. A variety of PerLum nanostructures are being fabricated and are frequently utilized in various fields, including bio-imaging, phototherapy, information storage, latent fingerprinting, and anti-counterfeiting, because of their distinctive optical qualities. With a higher signal-to-noise ratio (SNR) and increased sensitivity, PerLum-based bio-imaging may allow for guided cancer therapy [[11], [12], [13]]. Hence, such nanostructures represent a novel class of nanomaterials with so many benefits in biomedicine [14]. By using such materials, the background fluorescence and deep probing issues caused by excitation energies like UV–Vis, which are frequently used in luminescent imaging of different organisms, are eliminated [15]. Technologies that can perform therapeutics and diagnostics, called theranostic devices, have been brought about using PerLum nanomaterials. Currently, studies on therapies involving photo-thermal therapy [16], photodynamic therapy [17], gene therapy, and sensing have been stimulated by the quest for non-invasive and customized procedures for treatment. Photodynamic therapy (PDT) relies on the production of reactive oxygen species and singlet oxygen ions, which makes it a fascinating field of study that can render the use of traditional systems useless, thus allowing the use of PerLum materials in this technique of therapy [17]. Because there is no need for in-situ stimulation, PerLum nanomaterials possess a significant potential for biological imaging due to their extremely long decay times and excellent SNR. PerLum materials have proven excellent frameworks for developing multifunctional devices in imaging-guided drug distribution and theranostics when functionalized appropriately [18]. PerLum materials also have a lot of possibilities in fields related to informational technologies like information storage, latent fingerprinting, and anti-counterfeiting because of the extended reading window offered by their characteristics [19].

Matsuzawa et al. [8], marked the beginning of a renewed search for the underlying mechanism of PerLum, while until then, relatively little research was conducted on this subject. They described the mechanism of PerLum using the hole trapping and de-trapping model: when the Eu^2+^ ion is excited by an incident photon, a hole escapes to the valance band (VB), leaving behind an Eu^+^ ion, while the liberated hole is then captured by the co-doping Dy^3+^ ion, creating a Dy^4+^ ion. In the same year, Tanabe proposed a new model in which Eu^3+^ was created by leaving an electron in the conduction band (CB) while the Dy^3+^ ion captures the escaped electron through the CB and gets converted into Dy^2+^. This discovery prompted the search for more intense and long-lasting phosphors, and hence, 24 years after this breakthrough, many lanthanide-doped phosphors, with examples such as CaAl_2_O_4_: Eu^2+^, Nd^3+^ [20], Sr_4_Al_14_O_25_:Eu^2+^, Dy^3+^ [8] Sr_2_MgSi_2_O_7_:Eu^2+^, Dy^3+^ [21] and many others [22,23], have been fabricated to achieve this milestone. All such materials have found many uses in the current decade; for example, phosphors with blue and green emission colors have a vast range of civil applications and are readily available on the market [24]. In contrast to them, a very limited number of red and reddish-orange phosphors such as Y_2_O_2_S: Eu^3+^, Mg^2+^, Ti^4+^ [[25], [26], [27]], and (Ca_1-x_Sr_x_)S: Eu^2+^ have gained access to the market. For some particular phosphors, the afterglow has also been found to last for a couple of hours, with examples of CaAl_2_O_4_: Eu^2+^, Nd^3+^, and SrAl_2_O_4_: Eu^2+^, Dy^3+^ phosphors [8,20]. PerLum has a large number of applications ranging from luminous watches and clocks to medical diagnostics, night-vision surveillance, displays, in-vivo bio-imaging, and thermal sensors [8,[28], [29], [30], [31], [32]]. Sherman et al.*,* [33] reported in 2007 the use of PerLum nanostructures for real-time imaging in small animals for a duration longer than 1 h where, after in-vitro excitation, this phosphor emitted dark red NIR light and thus could be used as a promising contrast agent for a long time in-vivo optical imaging. Research on optical imaging, also known as fluorescence imaging, has rapidly grown in recent years with its application in brain imaging, sub-diffraction-limit imaging, molecular oncology, etc. [28,[34], [35], [36]].

**Fig. 2:**
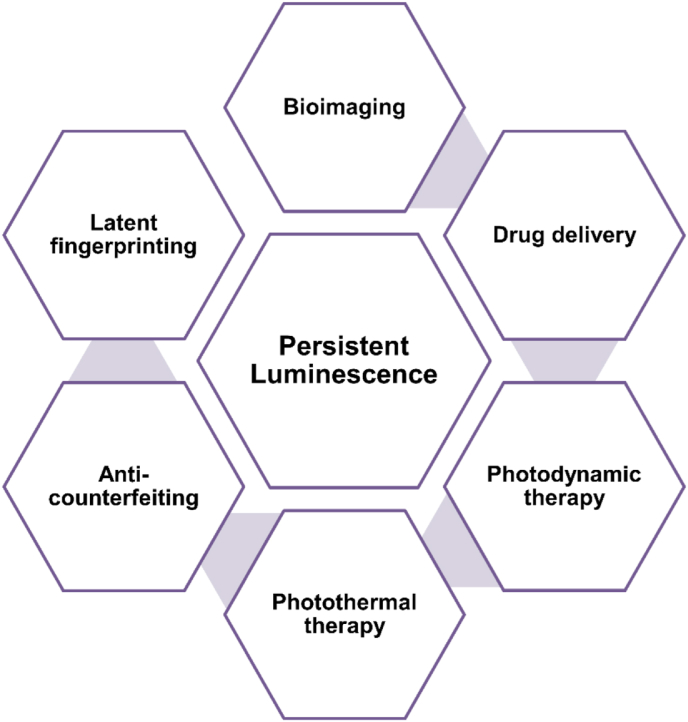
Illustration representing different applications related to long-lasting phosphors.

The PerLum property of a material is due to the presence of lattice defects in the host material. Energy sources such as UV light, X-rays, and visible light are used to generate electron-hole pairs, which are then trapped in trap centers generated by lattice defects and impurities [37,38]. These electron-hole pairs then recombine because of thermal stimulation, due to which PerLum is observed. Co-dopants such as transition and lanthanide metal ions are introduced to enhance the traps within the material, which can increase the intensity of PerLum by several orders of magnitude [8,39,40]. In this review, we discuss different aspects of PerLum starting from various mechanisms that have been used to explain this phenomenon, such as the VB and CB models, the Hoogeustraten model, the oxygen vacancy model, etc., along with different fabrication techniques used to synthesize such phosphors with such properties such as solid-state reactions, hydrothermal reactions, combustion reactions, etc. These materials, as already mentioned, show the emission of photons long after the excitation process is stopped due to the trapping of charge carriers. Thus, their property of lasting in emission for extended periods of time has earned them the name "long-lasting phosphor (LLP)" [41]. Several reviews have been published focused on the phenomenon of PerLum but lacking in some aspects of synthesis techniques, properties, or applications [19,24,[42], [43], [44], [45], [46], [47], [48], [49], [50], [51], [52]]. Thus, to counter this problem and provide up-to-date details related to such materials, this review provides detailed information regarding a variety of synthesis techniques, their advantages and disadvantages, as well as focusing on the various hosts used and different activators employed in them. It also has a special section dedicated to the emission and excitation characteristics of such materials, along with the properties of traps, which possess primary importance in the afterglow properties of PerLum materials. Because of the very detailed nature of this review, it is expected that it will act as a compiled source of information for the research community. The application part of this review also shows the importance of this field of study and will be helpful in providing enough motivation for future researchers. In this review, we will also focus on some frequently used PerLum materials with different activators used to improve their emission characteristics, along with some important properties of such LLPs. Finally, in this review, our focus will be on the biomedical and security applications of PerLum materials, as shown in Fig. 2. Over time, many attempts have been made to explain the phenomenon of PerLum. Although it is still not known with perfect certainty how this phenomenon exactly comes to be, some of the most prominent and accurate mechanisms used to explain PerLum have been explained.

## 2 Mechanism of PerLum

The mechanism of PerLum remained unexplored until the 1990s [8,20], thus no information was available until then, although ZnS:Cu materials had been well-researched and were available in the market [53]. During the study of this phenomenon, it was observed that some particular phosphor materials exhibit thermally stimulated PerLum at room temperature (∼20–25 °C or 293–298 K) when subjected to extremely high temperatures during their fabrication [54,55] The mechanism behind this phenomenon is still a topic of discussion [9,[56], [57], [58], [59]]. Research has shown that there are two types of activation centers: traps and emission centers [60]. Rare-earth (RE^3+^) ions such as Dy^3+^, according to Holsa et al. [61], may only serve as trap centers to get trap distribution via charge compensation. It was also found that by using Sm^3+^ as a co-dopant in SrAl_2_O_4_: Eu afterglow exhibited by this aluminate phosphor was improved as the RE co-dopant was observed to change the energy of traps while affecting the deeper traps more prominently. In almost all cases, the emission centers and trap centers have been found to be within the forbidden energy gap while being located more near the bottom of the CB with electron traps or slightly above the VB with hole traps, with a respective energy difference of approximately 1 eV or slightly more depending on the trap depth [54,55,57].

The trapping mechanism can be physically understood as a four-step process, (a) excitation of PerLum phosphor: where it is externally excited by high energy radiation such as ultraviolet, visible, or near-infrared, the afterglow phosphors release charge carriers (electrons or holes, or even both) if the external radiation has an energy greater than a certain threshold value. (b) The second step involves the storage of charge carriers at trap sites, which are then captured by electron and hole traps through the CB and VB, respectively, instead of being radiatively relaxed. These electron and hole traps do not radiate energy instantaneously but store it for an extended time, earning them the name "optical battery" [49,62]. The storage capacity of trapping states depends on the carrier concentration and defects present in the luminescent material. (c) In the third step, carriers are released from the trapping states after excitation has taken place. Once the excitation is halted, the carriers can start releasing charge because of mechanical [63,64], optical, or, in most cases, thermal excitation [[65], [66], [67], [68], [69]]. This process is called as de-trapping process. (d) The last step involves the recombination of excited charge carriers, where these charge carriers, after de-excitation, move back to the emission center, giving rise to PerLum owing to the recombination process. The properties of the emission center determine the frequency of emitted photons, while the intensity and duration of PerLum at a specific temperature are mainly determined by trap density, trap depth, and concentration of doping For the trapping and detrapping processes in persistent phosphor, trap types and trap concentrations, along with their depths, must be taken into consideration as they have a significant effect on the afterglow properties of a different material [56,57,69,70]. (1) Trap type: with LLP, the traps are generated because of lattice defects that can be intrinsic or may arise because of any added impurity [5,37,71,72] or because of high-energy radiation that may fall on the phosphor material [59,73]. The nature of traps generated by the above approaches is very hard to characterize, and it is thus very hard to decide which defect is responsible for the trapping of carriers. (2) The concentration of trap states is the second important aspect that plays a very important role for PerLum, as the probability of the charge carrier getting captured increases as the trap density rises. Enhanced electron reservoir potential is a significant task to enhance the afterglow in PerLum materials [72,[74], [75], [76]]. (3) The depth of traps governs the release rate of carriers captured in traps and at the same time as affects the intensity of PerLum and the afterglow time of phosphors post-excitation. At room temperature, shallow traps are drained quickly, thus generating intense phosphorescence that is assisted by the quick-release rate of charge carriers. Charge carriers stored in deep traps with a slow rate of release are barely drained at room temperature. The slow-release rate of these deeply trapped carriers results in a reduced intensity of phosphorescence and a long afterglow time [75,77]. In the case of RE dopants, the trapping mechanism is related to electron delocalization and tunnelling, where an electron is excited to the CB before it is delocalized into a trap [78]. This can be brought about by employing one of the three ways. (a) The dopant can be ionized by single-photon excitation to the 5d level, which is located inside the CB and from which the electron is finally sent to the CB The luminescence is completely quenched if all the 5d levels are inside the CB [79] (b) The RE-ion can be thermally ionized with the electron being sent to the CB if it is excited to the 5d level, just below the CB [80]. (c) Multiple-step photoionization can be employed to delocalize an electron to a trap if the 5d level is far below the CB [81,82].

### 2.1 VB and CB model

Trapping charge carriers is essential for the long-lasting afterglow of phosphorescence, and it is the fundamental process of energy storage in PerLum [72,[83], [84], [85]]. The trapping of such charge carriers is brought about by their delocalization near VB or CB, and when the charge carriers are excited, they get captured by the trapping state to be released later, after excitation. When the charge carrier is trapped in the VB or CB, it is free to move until its recombination or until it gets trapped again. According to this model, the CB and VB take part in excitation, delocalization through electronic bands, and the capture and release of charge carriers [52]. A good amount of research has been done on thermoluminescence glow curves and trap depths that point toward its popularity [54,86,87]. The discovery of SrAl_2_O_4_:Eu^2+^, Dy^3+^ by Matsuzawa and others marked the beginning of research on the mechanism of PerLum, and afterward, many attempts were made to explain this phenomenon, for example, the hole/electron model, quantum tunneling model, intrinsic defect model (also known as the oxygen vacancy model), etc. Pieter Dorenbos put forward the electronic level schemes of VB and CB of the host, energy levels of the ground, and excited states of activation centers. Because of his theory, it became possible to explain the phenomenon of PerLum and the process of trapping [[88], [89], [90], [91], [92], [93], [94]].

#### 2.1.1 Matsuzawa model

**Fig. 3:**
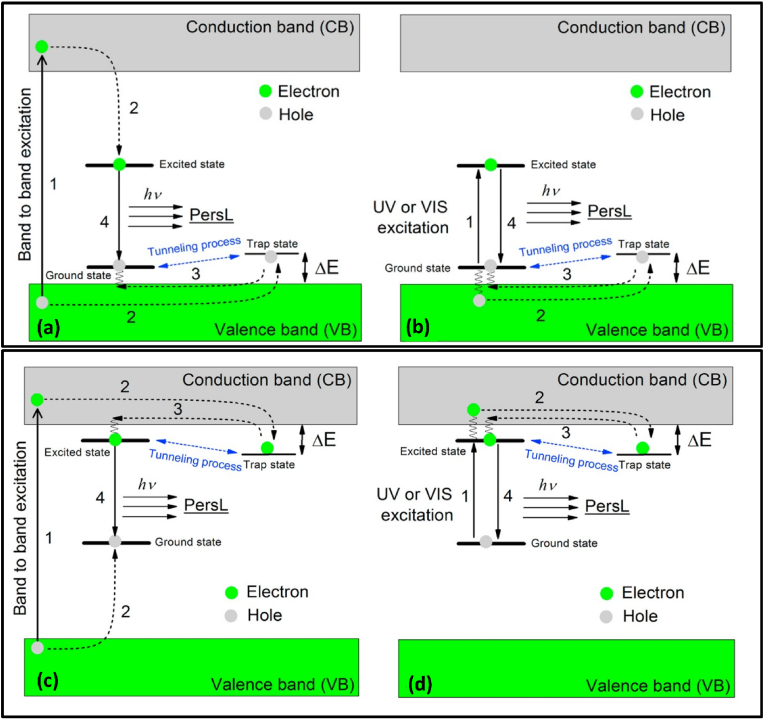
Illustration of the mechanism of PerLum based on trapping and de-trapping of a hole for (a) Band-to-band excitation with the help of high-energy photons (b) Pumping of emission ions with the help of low energy ultraviolet photons or visible photons. Reproduced with permission from Ref. [52], copyright 2019, Elsevier. (c) Illustration representing a mechanism of PerLum based on trapping and de-trapping of an electron in a trap state for (d) Band-to-band excitation with the help of high-energy photons (d) Pumping of emission ions by the help of low energy ultraviolet or visible light Reproduced with permission from Ref. [52], copyright 2019, Elsevier.

Also known as the hole model this model considers holes as the main charge carriers. This model was put forth by Matsuzawa et al., in a very famous research paper published in 1996, where they announced the discovery of Eu^2+^-activated SrAl_2_O_4_ phosphor co-doped with Dy^3+^ [8]. They based their assumption on results published by Abbruscato in 1971 about the optical and electrical properties of SrAl_2_O_4_ doped with Eu^2+^ [95]; there, it was proposed that the charge transfer mechanism in this sample involves the generation of a hole in VB, which can then recombine instantly or be trapped owing to lattice defects. Abbruscato further proposed that, in the sample used, almost 99 % of the total intensity is because of activation by the conduction of holes, thus considering holes as the main charge carriers [95]. Matsuzawa and others predicted that upon UV exposure (365 nm), Eu^2+^ excites because of the 4f to 5d transition. They argued that excitation does not occur for host material, and thus a hole is generated at the 4f ground state level, which is then thermally excited to the VB, and hence Eu^+^ is produced from Eu^2+^. They said that the hole thus generated is captured by Dy^3+^, which leads to the formation of Dy^4+^, thus trapping the hole, which is then released back to the VB after thermal stimulation. From the VB, it then recombines with the Eu ^+^ ion to form Eu^2+^; thus, de-excitation occurs along with the emission photon [8]. Thus, we can say that in the case of a hole, model holes are released from hole trapping centers, which recombine with electrons at the luminescent center to generate LLP. The limitation of this model is that it explains the phenomenon of PerLum in the case of doping such as Eu^2+^-Dy^3+^ and Eu ^+^ -Dy^4+^ in SrAl_2_O_4_ [8,[96], [97], [98], [99]], but it does not explain PerLum for SrAl_2_O_4_ with doping other than Eu^2+^ and Dy^3+^. Fig. 3 [52] give a schematic representation of the hole model to explain the phenomenon of LLP. If the hole model and electron model are analyzed correctly, then we can see that these two are mirror images of one another. In the case of the hole model, after using high-energy excitation (Fig. 3a) or direct pumping with the help of UV or visible photons (Fig. 3b), the electron is shifted to the CB, and the hole stays in the VB as a free charge carrier. The trap state then captured this free hole, while the electron gets shifted from the CB to the triplet excited state between VB and the CB. The ground state, located just above the VB, can share a hole with the trap state via the tunnelling process. The release of the charge carrier from the hole trap state is mediated by thermal energy, which recombines in the ground state to produce LLP [52]. The Matsuzawa model became quite famous in a short time [96,[100], [101], [102], [103], [104]] and has been often used to explain the phenomenon of PerLum [[105], [106], [107]]. Many experiments [20,101,[108], [109], [110], [111]] were performed to confirm this model, but the results did not comply with it. Thus, to explain the LLP of materials that do not follow the hole model, electrons were the primary charge carriers, and hence a new model was put forward known as the electron model.

#### 2.1.2 Aitasalo model

Also called the electron model, it considers the trapping of electrons as the reason for LLP. In the case of the hole model, which involves the trapping of holes that results in creating Eu^+^ and Dy^4+^ ions, which is not likely in the case of solids [9]. Thus, an alternate mechanism for PerLum was put forward by Aitasalo in 2003, which is based on the trapping of electrons rather than holes [9]. The electrons are directly excited from the VB to the trap level, and because of this process, a hole is also generated, which migrates to a calcium vacancy. It is important to mention that holes remain as free charge carriers in the VB, which explains the observations by Abbruscato and Matsuzawa used for their description of the PerLum of SrAl_2_O_4_:Eu^2+^, Dy^3+^ [20,95]. The electron is then transferred from the trap level to the oxygen vacancy with the help of thermal energy. Aitasalo argued that the oxidation of Dy^3+^ and reduction of Eu^2+^ (in the hole model) would generate ions that are chemically unstable [38]. The presence of cation and oxygen vacancies in calcium aluminates also provided support for the Aitasalo model [95,[97], [98], [99]]. As the CB is located high above the energy level of oxygen vacancies, a thermally assisted transfer of an electron from the VB to the CB occurs, which starts the recombination process.

**Fig. 4:**
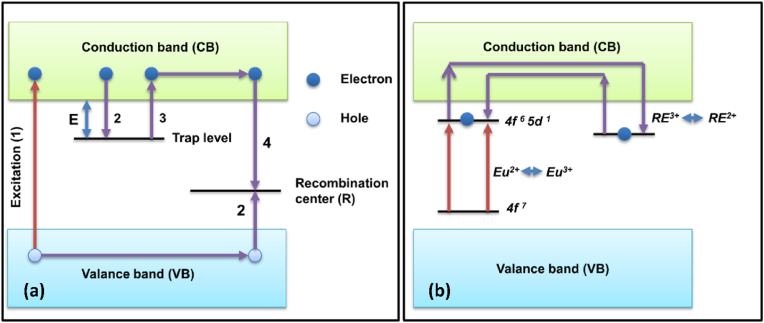
(a) Schematic diagram representing the process of thermal assisted release of trapped charge Carriers divided into 4 steps (1) excitation by a suitable wavelength (2) trapping of an electron in trap level and shifting of a hole to recombination center (3) release of charge carrier to CB triggered by thermal energy and (4) recombination of electron and hole at recombination center which results in the production of luminescence [55]. (b) Schematic representation of the mechanism of PerLum as proposed by Dorenbos to explain the LLP in the case of aluminates and silicates.

According to this model, either the traps are directly supplied with the excitation energy or this energy is provided through the CB, and owing to this process, the electrons are trapped in the oxygen vacancy. As the energy level of the oxygen vacancy trap is located below the CB, it allows the thermally assisted transfer of electrons to the CB, thus starting the electron-hole recombination process. The recombination mediates the excitation of Eu^2+^ via non-radiative transition, and according to Aitasalo, this process uses close contact between defect centers such as oxygen and calcium vacancies and the luminescent centers. The energy produced by the recombination process excites the europium to the 5d level [112], which is followed by the recombination process and by PerLum [9]. Fig. 3 [52] show the schematic representation of the electron model used to explain the LLP. In Fig. 3c, a high-frequency energy source (a mercury UV lamp) has been used for excitation, while Fig. 3d explains the pumping of the emission center with the help of low-frequency photons, such as low-energy UV photons. By absorbing energy from high-frequency photons, electrons make a transition from VB to CB, leaving behind a hole, and thus both of them are free to move in their respective bands. Afterward, the electron is trapped by the trap center while the ground state of the emission center traps the hole, and thus the process of excitation is completed. After completion of the excitation process, the charge carrier, which has already been captured, is released with the help of thermal stimulation. The probability of the release of the charge carrier from the trap level is directly related to the energy gap between the bottom of the CB and the trap level denoted by E, as the release of the charge carrier from the trap level is only possible if energy gained due to thermal energy is equal to or greater than ‘E," satisfying the condition as shown in Fig. 4a. Because of the recombination of an electron released from the trap site and the hole present in the VB, a PerLum is generated. If the excited electron is trapped due to intrinsic defects that are very shallow, then it can be released within very little time, which gives rise to an afterglow between μs or ms [[113], [114], [115], [116], [117]]. Because of thermal stimulation, the probability of release of the charge carrier from the trap site per unit of time is given by the Arrhenius equation:

(1) $$p=s\exp\left(-\frac{E}{KT}\right)$$

The term ‘s’ represents frequency factor or attempt to escape factor which is temperature independent with the value ranging from 10^12^ – 10^14^ s^−1^.

In the year 1996, Tanabe also proposed that Eu^3+^ is created post-excitation when an electron shifts to the CB, which is then captured by Dy^3+^ to form Dy^2+^. This theory got strong support from the fact that Eu^3+^ was observed in afterglow materials after the analysis of X-ray absorption near the edge structure (XANES). The co-dopant Dy^3+^ ion in the electron model is considered to be contributing to defects and also increasing the oxygen vacancy in SrAl_2_O_4_. The increase in the number of lattice defects is because trivalent lanthanide ions occupy the alkaline earth sites, which are divalent, which leads to defect creation because of the charge compensation process [38]. The addition of Sm^3+^ to increase PerLum has the same explanation of charge compensation as it is reduced to Sm^2+^ during preparation, thus removing hole vacancies, such as cation vacancies. Holsa et al. [9], also proposed that Eu^+^ and Dy^4+^ were unstable in aluminate or silicate compounds, thus supporting the hole model. They also argued that by adding a RE dopant, it is possible to modulate the trap state with the help of charge compensation. They found that the addition of Sm^3+^ ions to SrAl_2_O_4_:Eu proved to be an important factor affecting the duration of afterglow in this phosphor as the Sm^3+^ ion gets reduced to Sm^2+^ easily in the N_2_–H_2_ atmosphere, which leads to a decrease in the concentration of traps. Based on this phenomenon, reasonable explanations were proposed for the mechanism of LLP for CaAl_2_O4: Eu co-doped with Nd, and for ZrO_2_: Ti. This group employed techniques such as EPR, XPS, and XANES, which proved oxidation of Eu^2+^ to Eu^3+^ during the process of excitation, while during experimentation no change was observed in the valency of co-dopants. It was observed that when a host was doped with two dopants instead of one, they showed a strong afterglow that lasted for a longer duration. The reason for this stronger and longer afterglow is that the co-dopant helps to regulate trap parameters [118]. The mechanism of PerLum proposed by Matsuzawa was not accepted by Clabau et al. [119], as they observed a decrease in the concentration of Eu^2+^ ions during excitation in EPR measurements, but as soon as excitation ended, their concentration was seen to increase, which continued until luminescence ended. Thus, they proposed that Eu^2+^ helps in the process of trapping and de-trapping, which is opposite to the idea of energy transfer to Eu^2+^ after a charge carrier is trapped.

#### 2.1.3 Band engineering model (dorenbos model)

The electron model was accepted by Dorenbos and coworkers, but they argued that the electron model presented by Aitasalo considers that after excitation, the charge carrier remains in the ground state of Eu^2+^, which is a wrong assumption [120]. Because, in the case of lanthanides, energy levels are localized, in contrast to the Bloch states of VB and CB which are delocalized, thereby Dorenbos and others proposed a different mechanism of LLP in 2005 [90]. They expected electrons to be excited by Eu^2+^ ions in the VB and thus convert them into Eu^3+^ ions. As the 5d energy state of the Eu ion is located very near to CB, excited electrons can easily shift to CB, after which they combine with a trivalent RE-ion to give rise to a divalent RE-ion, as shown in Fig. 4b [19]. This model of PerLum also faced the limitation of not being able to explain LLP with SrAl_2_O_4_:Eu^2+^. This group proposed several approaches to calculate energy levels for different phosphors to explain their PerLum, for which it is very essential to contemplate some properties like the location of energy levels of RE^2+^ and RE^3+^-ions, the energy band gap of the host material used, and the calculation of the position of the trap level regarding the bottom of the CB of the host material. The location of trap states can be estimated with the help of thermoluminescence spectroscopy, and the energy band gap could be calculated using diffuse reflectance spectra, but the tricky part is calculating the location of the energy levels of RE-ions, i.e., RE^2+^ and RE^3+^. By taking the example of CdSiO_3_ doped with Tb^3+^, Holsa, and others predicted a method for calculating the ground state excitation energies of RE^2+^ and RE^3+^-ions [[121], [122], [123]]. They proposed that according to the previously discussed mechanisms, the energy at ground level for RE^3+^-ions has been independent of the host, so once the ground state energy of any RE^3+^-ion has been calculated, that data can be used to calculate the ground-level energy of any other RE-ion with the help of Dorenbos's model of PerLum. They also proposed that it is very important to consider the intensity of the transition to generating accurate data to explain the reason behind LLP. Based on this theory, the ground state energy levels for Sr_3_MgSi_2_O_8_ were calculated, and the best possible RE-ions to act as emission centers were estimated to be Eu^2+^, Ce^3+^, Tb^3+^, and Pr^3+^. These ions produce strong emissions and also possess a very low tendency for cross-relaxation and multi-photon de-excitation. One of the main requirements of LLP is that we should be able to excite a nanophosphor with a photon from the visible range, which is present in normal daylight, or with the help of UV light possessing the lowest possible energy. Now, as far as Eu^2+^, Ce^3+^, Tb^3+^, and Pr^3+^ are considered, they possess the lowest 5d energy, so they can be easily excited with the help of low-energy photons such as low-energy UV or even blue light with Eu^2+^. By using such atoms as dopants, overlap between the 5d excited level and the CB can also be ensured, so that transferring an electron to the trap level becomes efficient and easy [[124], [125], [126]]. The glow curves of YPO_4_:Ce^3+^, RE^3+^, and YPO_4_: Pr^3+^, RE^3+^ were studied by Bos et al. [127], and Lecointre et al.*,* [128]^,^ respectively. The RE-used in the former case were Nd, Sm, Dy, Ho, Er, and Tm, and in the latter case, the RE-ions used were Nd, Dy, Ho, and Er. It was observed that the thermoluminescence peaks obtained in both cases matched each other perfectly, and the values of electron activation energy have been observed to be the same for both of these phosphors. This observation predicted that the addition of co-dopant plays a role in the trapping process when, typically, it is not its property.

#### 2.1.4 Clabau model

**Fig. 5:**
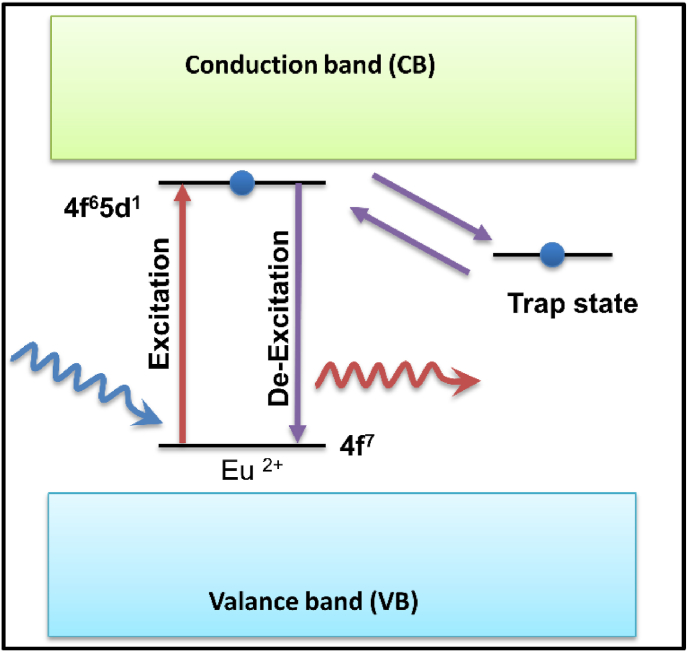
Schematic diagram explaining the phenomenon of PerLum as proposed by Clabau and coworkers for strontium aluminate doped with Eu^2+^ and Dy^3+^.

Clabau and others also analyzed the already proposed mechanisms of PerLum and concluded that they could not explain this phenomenon with perfect accuracy and needed a modification. They also didn't accept the hole model proposed by Matsuzawa for the same reasons as those put forward by Dorenbos. They further added that EPR measurements indicate that there is a fall in the concentration of Eu^2+^ during the process of excitation and then a rise in its concentration after the source of excitation is switched off, which keeps on increasing until the afterglow continues. Thus, they concluded that this fall and rise in the concentration of Eu^2+^ must be because this ion takes part in the trapping process, which contradicts the process of energy transfer to Eu^2+^ after trapping as proposed by Aitasalo [119,129,130]. The model of PerLum proposed by Clabau et al. [119], as shown in Fig. 5 [57], was almost the same as the one proposed by Dorenbos, with some important modifications, such as the fact that in this model, Clabau et al. [119], considered that there was no migration of electrons via the CB but that transferring electrons was rather a direct process between the trap state and a luminescent center. Shifting of electrons between the trap state and luminescent center requires proximity of lattice defects with Eu ions, as shown by the results of photoconductivity measurements of strontium aluminate doped with Eu^2+^ and Dy^3+^ and excited with the help of UV photons. During this measurement, it was observed that up to a temperature of 250 K, the value of photoconductivity increases, which attains its peak value at a temperature of 300 K. Getting the peak value of photocurrent means that there are no more free charge carriers that could be released at this temperature, but thermoluminescence measurements show that at 300 K the process of de-trapping is going on, which led Clabau and others to conclude that interaction between the trap state and luminescent center does not take place via CB. Another important point that Clabau and others noticed was that by comparing the glow curves of undoped SrAl_2_O_4_ and SrAl_2_O_4_: Eu^2+^ co-doped with Dy^3+^, the peaks of these two glow curves were found to vary in size and location, but have a similar shape, which led them to conclude that the chemical nature of the trap does not vary with the addition of a co-dopant. Thus, they concluded that oxygen vacancies act as traps in strontium aluminate doped with Eu^2+^ and co-doped with RE^3+^ [129]. Using lanthanide co-dopants was found to assist in stabilizing the oxygen vacancies because of their lower ionization potential, and a lower value of ionization energy will attract oxygen vacancies more strongly, thus increasing the depth of traps [130]. Such results imply that if the RE co-dopant ion has a higher ionization potential, then the time of afterglow will decrease [119].

**Fig. 6:**
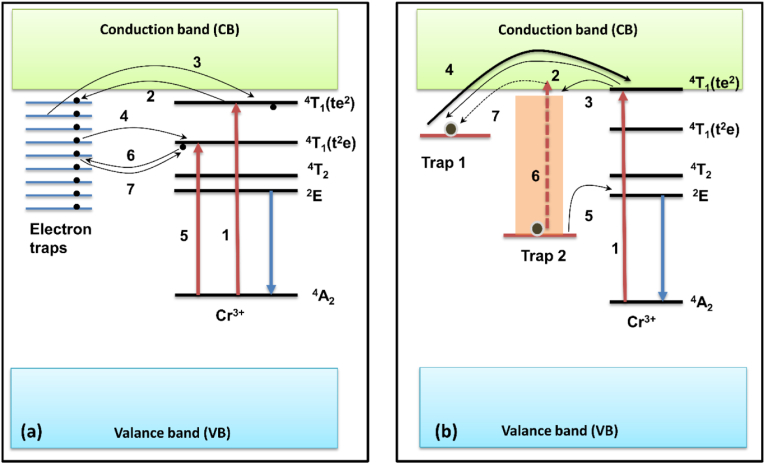
(a) schematic diagram representing the mechanism of near IR Per Lum. In the above figure, straight lines with arrowheads represent optical transition while curved lines with arrowheads represent the process of electron transfer. (b) figure representing the mechanism of near IR Per Lum and photo stimulated Per Lum in the near IR range.

### 2.2 Hoogeustraten model

This model is also known as the quantum tunnelling model, which refers to a process where a particle can overcome a barrier that is not possible for it to do classically. This process takes place near the excited energy levels of activator ions and trap states. With the aid of this model, PerLum can be explained because of deep traps, as this model does not require any physical proximity of trap levels with VB or CB. This model was proposed by Hoogeustraten and others [131] in 1958. After this Tanabe and coworkers [132] also proposed a similar model to explain LLP for strontium aluminate doped with Eu^2+^ and Dy^3+^. By proceeding with the measurement of photocurrent, Tanabe and others found that the maximum number of 5d energy levels of the Eu^2+^ ion, which replaces Sr in the host, has a lower crystal field residing inside the CB. Based on this finding, they concluded that the trapping and de-trapping processes occur via the process of quantum tunnelling, which is in contrast with the idea that trapping and de-trapping occur via VB and CB. In the year 2012, Pan et al. [133], studied zinc gallogermanate phosphors with strong emission in the wavelength range of 650 nm–1000 nm and a very long afterglow of approximately 360 h.

Variations in the energy used in activation proved that the threshold ionization energy level in Zn_3_Ga_2_Ge_2_O_10_ lies very near to CB. These findings were very similar to the VB-CB model as represented in processes 2 and 3 of Fig. 6a [19] and process 2 and 4 in Fig. 6b [19]. During their study, they found that visible light can also produce LLP with a very weak intensity but an extra-long afterglow, because of which they proposed that another mechanism of LLP was at play in this nanophosphor, which involves the trapping and de-trapping of electrons. This mechanism of PerLum is called the quantum tunnelling model and is explained by processes 4, 6, and 7 of Fig. 6a [19] and by process 5 of Fig. 6b [19]. With the help of visible light excitation, electrons present in the ground state of the Cr^3+^ ion shift to deep traps with an energy lower than the threshold ionization energy. Trap states are further filled by the tunnelling process from Cr^3+^ energy levels whose energy matches that of trap states. Because of the reverse tunnelling process, PerLum with a weak and super-long afterglow is produced. The near-IR PerLum of LiGa_5_O_8_ also supported all these proposed phenomena doped with Cr^3+^ with an afterglow of 1000 h [134].

### 2.3 Oxygen vacancy model

Yang et al. [19], proposed this mechanism of PerLum based on the study of luminescence triggered by photons from fluoro-aluminate glasses doped with Eu^2+^ [135], alkali-doped silicate and borate glasses doped with Ce^3+^ [136], strontium aluminosilicate glasses doped with Eu^2+^ [137], and glasses doped with gold nanoparticles [138]. By studying EPR spectra, they explained the trapping and de-trapping of charge carriers based on the model of PerLum called the oxygen vacancy model. Oxygen vacancies are stable and can explain PerLum in oxide-based hosts. In the case of the oxygen vacancy model, such vacancies act as traps for electrons and thus lead to an extended afterglow. One of the main advantages of this model of PerLum is the confirmation of such trap types instead of the theoretical approach of the VB/CB model and the quantum tunnelling model. Oxygen vacancies have been mentioned as one of the main reasons for PerLum, as Aitasalo and others considered them very important traps in their model to explain PerLum [9] in the case of strontium aluminate doped with Eu^2+^ and Dy^3+^, and Clabau and others also mentioned them as highly efficient traps for electrons [119]. As mentioned earlier, by comparing the glow curves of SrAl_2_O_4_ doped with Eu^2+^ and SrAl_2_O_4_ doped with Eu^2+^ and co-doped with Dy^3+^, it was found that the thermoluminescence peaks of these two phosphors varied in size and position but had similar shapes. From this study, they proposed that the use of co-dopants does not affect the chemical nature of traps, and thus only oxygen vacancies assist in the trapping and de-trapping of electrons for LLP. Lim et al. [139], studied spinal phosphors doped with Ti and concluded that red emission in the wavelength range of 620 nm is due to Mg^2+^ and O^2−^ vacancies. They based this finding on the fact that in the case of pure spinal intrinsic defects known as Schottky defects, such as Mg^2+^ and O^2−^ vacancies, they were present in abundance and had also appeared in pairs, thus supporting the oxygen vacancy model.

It can be said that for the generation of efficient luminescence along with extended afterglow, it is very essential to choose a suitable host along with an appropriate dopant and co-dopant. Over time, many hosts have been synthesized and afterward doped with different activators, mostly RE-ions, along with a few transition and main group elements as well. The quest to find more and more efficient PerLum nanostructures with improved luminescent and structural properties along with extended afterglow is still going on, with quite a few researchers shifting their field of study in this direction. One of the most prominent reasons behind this is the vast field of applications for such materials to be used in fields like bio-imaging, therapy, sensing, security, glow-in-the-dark signs, toys, etc.

## 3 Components of persistent luminescence nanophosphors

The selection of a suitable host and activator is very important for a phosphor to display the phenomenon of PerLum. The host acts as a trap carrier during activating LLP, and because of the different properties of traps, a variety of hosts may possess varying storage abilities for charge carriers. A host can possess two types of defects, and both of them aid in producing traps for charge carriers. These defects include intrinsic thermal defects, which are present in the lattice structure of the host, and extrinsic defects, which are introduced via different phenomena, such as the addition of a dopant, *etc.,* [140]. Some particular characteristics of a PerLum nanophosphor host and dopant play a very important role because the process of luminescence is based on energy absorbed by the host lattice or dopant ions, the outcome of which is absorption and consequent emission of energy [141]. Luminescence involves excitation with the help of a suitable excitation source and the emission of visible light, which is commonly caused by an impurity ion, also called an activator ion. In some particular cases, if emission from an activator ion is too weak, a co-dopant is added to the host material. Such an added impurity, which may act as a sensitizer, possesses the property of strong energy absorption, and this absorbed energy is quickly transferred to the activator site thus, enhancing luminescence properties. Hence, for efficient luminescence and color tunability, the choice of a suitable host and dopant is very important [142,143]. In this part of the review, we will discuss the classification of host materials along with the variety of hosts used to generate PerLum.

### 3.1 Hosts

**Fig. 7:**
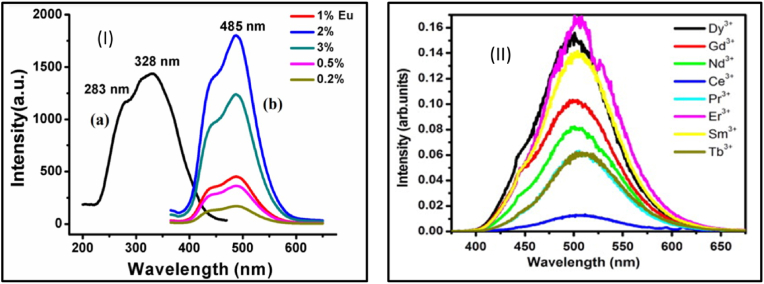
(I) Photoluminescence spectra of BaAl_2_O_4_ doped with Eu^2+^ (a) Excitation spectrum. (b) Emission spectrum showing the effect of Eu^2+^ activator ion. Reproduced with permission from Ref. [148], copyright 2017, Springer Nature (II) Emission spectrum of BaAl_2_O_4_:Eu^2+^ co-doped with different lanthanide ions to observe the effect of RE co-doping on luminescence spectrum. Reproduced with permission from Ref. [149], Copyright 2012, Elsevier.

As already discussed, the choice of a suitable host and activator is very important for extended afterglow and strong emission. Trapping charge carriers leads to the phenomenon of PerLum, so the availability of trap states is imperative. In a PerLum nanomaterial, traps are introduced via defects that have already been mentioned as intrinsic and extrinsic. Intrinsic defects can be of many types, such as cation vacancies, anion vacancies, thermal defects, and interstitial ions. Most of these defects are brought about by high-temperature treatment, so the volatilization temperature of the metals and metal oxides must be taken into consideration. In the case of oxides, the formation of traps has been observed in hosts whose melting point is low and volatility temperature is high, for example, ZnO, SrO, WO_3_, CaO, P_2_O_5_, and GeO_2_, but the formation of traps has also been seen with Al_2_O_3_, SnO_2_, Ga_2_O_3_, etc., which possess a low volatility temperature that allows faster volatilization of metal ions. Apart from the fabrication technique used and the condition of synthesis, the crystal structure of the host and the location of the activator in this crystal structure also play a very crucial role in generating afterglow. For example, with ZnGa_2_O_4_, which possesses an AB_2_O_4_-type spinal structure where A represents zinc ions and B represents gallium ions occupying tetrahedral and octahedral sites, respectively, a very peculiar property is observed in it, i.e., this spinal structure shows slight inversion characteristics in which a few zinc and gallium ions exchange their positions, thus giving rise to a crystal defect known as an anti-site defect. It has been observed that this compound displays an inversion of approximately 3 %, so almost 3 % of zinc and gallium ions exchange positions. It has also been observed that when a gallium ion takes the place of a zinc ion, it generates a defect with a positive charge, whereas when zinc replaces a gallium ion, a negatively charged defect is generated [144,145]. Li and others [146] took advantage of the inverted spinal structure property by synthesizing Zn_2_SnO_4_ and doping it with Cr, where they employed the easy replacement of Zn^2+^ and Sn^4+^ by Cr^3+^ ions, which gives rise to distorted octahedral-type coordination and unequal substitution. In the comparison of normal structure, the inverse spinal structure provides a different arrangement of cations, where the structure of ZnGa_2_O_4_ involves Sn cations and 50 % of zinc cations are located at octahedral sites while the other 50 % of Zn cations occupy tetrahedral sites. Because of this type of structure, despite having zinc vacancies and interstitials, it also contains some anti-site defects, which are generated when zinc and tin ions replace their positions, and hence it could be predicted that some unequal substitutional defects would be formed [147]. Apart from these intrinsic defects, some extrinsic defects can also be generated by introducing dopant ions. Research in PerLum has shown that doping can vary the properties of traps such as distribution and density along with increasing depth and concentration of traps, e.g., with SrAl_2_O_4_ doped with Eu^2+^, the duration of the afterglow is very short and emission intensity is also weak, but with the use of co-dopant Dy^3+^, the afterglow time increases while increasing the intensity of emission at the same time. Using co-dopant does indeed allow the generation of defects in the lattice structure, but it is also closely related to the properties of dopant, viz., atomic radius, doping concentration, valance state, etc. Gedekar et al. [148], studied the luminescence of Eu^2+^-doped BaAl_2_O_4_ and, after studying the PL spectra of this nanophosphor, they observed that the excitation spectrum featured three peaks at 270, 328, and 397 nm, as shown in Fig. 7 (I) [148]. It was observed that the 328 nm peak was five times more intense than that of 270 nm peak. This excitation spectrum was attributed to the 4f^7^ to 4f^6^5d transition of the activator ion. They observed that the emission spectrum for this nanophosphor does not seem to depend on the choice of wavelength used for excitation and features two distinct peaks at 485 and 433 nm. Maximum emission was seen to be centered at 485 nm, which was attributed to the 4f^6^5d to 4f^7^ transition emitting blue light. Lephoto et al. [149], also synthesized BaAl_2_O_4_:Eu^2+^ via the combustion method and studied the effect of using a co-dopant among the lanthanide series. They used photons in the wavelength range of 325 nm for excitation, and PL studies of this nanophosphor showed maximum intensity for co-doping of Eu^3+^ while the least intensity was observed for Ce^3+^ co-doping, as shown in Fig. 7 (II) [149]. They also observed that only Nd^3+^ and Dy^3+^ showed an afterglow of longer duration, and the same was also observed by Katsumata et al. [150], who used co-doping of Nd^3+^ and Dy^3+^ in MAl_2_O_4_:Eu^2+^.

It has been seen that in most PerLum nanomaterials, intrinsic defects induced by thermal energy along with extrinsic defects collectively affect the duration and intensity of the afterglow. The optical properties of ZrO_2_:Ti^3+^ and ZrO_2_:Ti^3+^ co-doped with Lu^3+^ were studied by Holsa et al. [151], and they found that Ti^3+^ acted as the center of luminescence while introducing Lu^3+^ improved the afterglow time. They explained this phenomenon via the mechanism of the generation of oxygen vacancies, where they stated that the Lu^3+^-ion settles in the location of Zr^4+^ in ZrO_2_: Ti^3+^, Lu^3+^, which results in the generation of oxygen vacancies via the phenomenon of charge compensation. These oxygen vacancies act as a reason for a change in trap distribution for the host, thus acting as traps for electrons and holes in the lattice structure. Such types of extrinsic and intrinsic thermal defects were also observed in the lattice structure of Sr_2_MgSi_2_O_7_: Eu^2+^ and co-doped with RE^3+^ [152]. It has been observed that in this phosphor, intrinsic defects involve oxygen vacancies, cation vacancies, as well as interstitial ions. The existence of cation vacancies is due to the replacement of RE^3+^ by a metal ion with a charge of +2. In many other PerLum nanomaterials, such as CdSiO_3_, etc., almost the same distribution of traps has been found while they are undoped or when they are doped with RE^3+^ ions. The reason behind such trap distribution is considered to be oxygen and cadmium vacancies, which result from CdO evaporation. Charge compensation defects result from the replacement of Cd^2+^ by RE-ions, which give rise to interstitial oxide ions and cadmium vacancies [121].

### 3.2 Activators

The phenomenon of PerLum also largely depends on the properties of emitter centers, as after absorbing energy, they should be able to emit visible radiation, which generates an emission spectrum. In nanophosphors, there are many ions used as activators, but for PerLum examples of ions used as activators, there are very few, which include some RE-ions (Ce^3+^, Sm^3+^, Eu^2+^, Eu^3+^, Tb^3+^, Dy^3+^, Yb^2+^, Yb^3+^, etc.), transition metal ions such as Cr^3+^, Mn^4+^, Mn^2+^, Ti^4+^, etc., and some elements of main groups such as Bi^3+^, etc. In this review, we will discuss different aspects of PerLum related to RE-ions, transition metal ions, and some elements in the main group.

#### 3.2.1 Rare earths as activators

RE-doped PerLum has received a lot of attention over the past few years and has been extensively used in luminescent materials. One of the main advantages of the use of RE-ions as activators is that they allow excitation by almost any kind of energy source. In most cases, the efficiency of luminescence decreases sharply in the presence of lattice defects, but with PerLum nanomaterials, defects allow for an increased duration of afterglow as the defects act as traps for electrons and holes [9]. Starting in the mid-1990s, a completely different type of PerLum nanophosphors with RE-ion doping were synthesized and studied [153], such as nanophosphors having a general formula of MAl_2_O_4_ doped with Eu^2+^ (M = Ca, Sr) [154,155]. Apart from these nanophosphors, some other LLPs with more complex lattice structures and RE-doping were also studied [[156], [157], [158]].

One of the most famous dopants used for PerLum is Eu^2+^, which is used as an activator ion and whose emission color varies depending on the electrostatic field of surrounding ions (also called the crystal field effect). With phosphors such as SrAl_2_O_4_:Eu^2+^ co-doped with Dy^3+^ [159], Sr_2_MgSi_2_O_7_:Eu^2+^ co-doped with Dy^3+^ [160] and CaAl_2_O_4_:Eu^2+^ co-doped with Nd^3+^ [20] etc., blue emission is obtained once they are excited with a suitable energy source. Some other nanophosphors display green emission once excited with photons of suitable frequency, for example, Ba_2_MgSi_2_O_7_:Eu^2+^ co-doped with Tm^3+^ [161], SrAl_2_O_4_:Eu^2+^ co-doped with Dy^3+^ [20], etc. For emission to appear in the colors yellow, red, or orange, it is very important that the strength of the crystal field be such that it can lower the lowest energy state of the 4d^6^5 d^1^ configuration. Some of the potential candidates that show this kind of emission are Sr_3_SiO_5_:Eu^2+^ co-doped with Lu^3+^ [162,163], Sr_2_Al_2_Cl_2_O_5_:Eu^2+^ co-doped with Tm^3+^ [164], Ca_2_BClO_3_:Eu^2+^ co-doped with Dy^3+^ [7], etc. PerLum of oxides is triggered with the help of UV light, while Eu^2+^-doped silicates and nitrides show emission falling in the wavelength region of red light. RE-doped nanophosphors have also been observed to emit NIR PerLum, so they can be used for biological applications. For example, the red light-emitting phosphor BaMg_2_Al_2_N_4_:Eu^2+^ co-doped with Tm^3+^ synthesized by Ueda et al.*,* [165] emits NIR PerLum. This luminescent material could be charged using red light and could be used for imaging biological subjects in the first biological window. Currently, research in the field of PerLum is mainly focused on aluminates and silicates, as it has been observed that the afterglow emission from such nanophosphors is strong and the afterglow time is also sufficiently long [166]. One other very important ion used as an afterglow activator is Ce^3+^, which possesses an electronic configuration of 4f^1^. Because of this property, it can display broad emission, as the 4f^1^ configuration ensures a strong crystal field, whose strength depends on the transition energy of the 5d-4f transition [167]. Some examples of PerLum nanophosphors with an afterglow of more than 3 h and blue-green emission are Ca_2_Al_2_SiO_7_ doped with Ce^3+^ [168], Lu_2_SiO_5_:Ce^3+^ [169], SrAl_2_O_4_:Ce^3+^ [170,171], CaAl_4_O_7_: Ce^3+^ [56,57], etc. Two important characteristics of nanophosphors doped with Ce^3+^ are that the most commonly used hosts from aluminate family and that the emission for such PerLum nanomaterials lies in the wavelength range of the blue region. Some examples of PerLum nanomaterials that display afterglow in the wavelength range of green and yellow are: Y_3_Sc_2_Ga_3_O_12_ doped with Ce^3+^ with bluish green emission [172,173], Lu_3_Al_2_Ga_3_O_12_:Ce^3+^ with bluish green emission [151], Y_3_Al_2_Ga_3_O_12_:Ce^3+^ with green emission [174] etc. In most nanophosphor wavelengths of photons used for excitation lie in the range of blue light, while UV light is also being used for most of the PerLum nanomaterials for exciting them to a higher energy state.

Using Eu^3+^ as an activator has also been studied in good detail because of its property of efficient red emission, which is due to a large number of transitions from the ^5^D_0_ excited state to the ^7^F_J_ (J = 0, 1, 2, 3, 4) energy levels of the 4f^6^ configuration, with the wavelength of all these transitions falling in the red-orange region of the electromagnetic spectrum. A large number of oxy-sulfide-based nanophosphors doped with Eu^3+^ have been studied, such as Gd_2_O_2_S: Eu^2+^ co-doped with Mg^2+^ and Ti^4+^ [175], Y_2_O_2_:Eu^2+^ co-doped with M^2+^ and Ti^2+^, where M denotes metals from the group of alkaline earth metals such as Mg, Ca, Sr, Ba, and Y_2_O_2_S: Eu^3+^ co-doped with Zn^2+^ and Ti^4+^ [176,177]. In case of a host such as Y_2_O_2_S and Gd_2_O_2_S, Eu^3+^ used as an activator replaces Y^3+^ and Gd^3+^ and thus acts as a center of emission. Co-dopants such as Mg^2+^ and Ti^4+^ used in these phosphors also replace the same sites in their respective hosts and then act as sensitizers by absorbing energy and then transferring that energy to the activator or emission center. Apart from oxy-sulphides, a large number of oxides doped with Eu^3+^ have been reported to show the properties of PerLum, viz., SrMg_2_(PO_4_)_2_: Eu^3+^ co-doped with Zr^4+^ [178], Lu_2_O_3_ doped with Eu^3+^ [179], Ba_3_Gd_8_Zn_4_O_21_ doped with Eu^3+^ [180,181]^,^ etc. Sm^3+^ is also a widely used activator because of its strong red emission, which is attributed to the transitions between the ground and excited electronic states of this activator. Because of its property of intense red emission, Sm^3+^ has been used as an activator in a large number of LLPs that involve oxides, oxy-sulphides, and fluorides such as La_2_Zr_2_O_7_:Sm^3+^ and co-doped with Ti^3+^ [182], CaO doped with Sm^3+^ [183], La_2_O_2_S doped with Sm^3+^ [184], KY_3_F_10_ doped with Sm^3+^ [185]^,^ etc., were studied in detail. To study the afterglow properties of Sm^3+^, a large number of stannate-based compounds have been doped with this activator, viz., Ca_2_SnO_4_ [186], Sr_2_SnO_4_ [187], CaSnSiO_5_ [188] etc. For the nanophosphor Sr_3_Sn_2_O_4_ doped with Sm^3+^, excitation due to ultraviolet light results in strong emission of reddish-orange light attributed to ^4^G_5/2_ to ^6^H_J_ (where J take values 5/2, 7/2, and 9/2). The afterglow from such transitions has been observed to be very bright and visible to the naked eye for an hour [189]. Similarly, UV (254 nm) excitation of Na_2_CaSn_2_Ge_3_O_12_ doped with Sm^3+^ results in the emission of photons in the red wavelength range. Afterglow of this emission has been observed to last for 4.8 h, ascribed to a transition from its ground state, i.e., ^4^G_5/2_ to its lower energy states [190]. Another activator that displays red emission with chromaticity coordinates of (0.68, 0.31) is Pr^3+^, with emission attributed to transitions of ^1^D_2_ to ^3^H_4_, ^3^P_0_ to ^3^H_6_ and ^3^P_0_ to ^3^F_2_. For the first time, a red afterglow among Pr^3+^-doped compounds was found in perovskite-type oxides with a chemical formula of ABO_3_, such as CaTiO_3_ [191] NaNbO_3_ [192] and SrZrO_3_ [193] etc. After this, many other PerLum nanomaterials were reported which were observed to emit photons in the wavelength range of red region, viz., CdGeO_3_ doped with Pr^3+^ [194], Y_3_Al_5_O_12_ doped with Pr^3+^ [195], ZnTa_2_O_6_ doped with Pr^3+^ [196], La_2_Ti_2_O_7_ doped with Pr^3+^ [182], Ca_2_SnO_4_:Pr^3+^ [197], etc. Strong emission in the wavelength range of green has been obtained by the use of Tb^3+^ as an activator, attributed to transitions from ^5^D_4_ to ^7^F_J_ (J takes values of 6, 5, 4, 3). This green emission resulted from the radiative relaxation of the excited Tb^3+^ ion, corresponding to transitions from the ^5^D_4_ state to the ^7^F_J_ state. The longer afterglow is because of the process of cross-relaxation between two adjacent Tb ions, which leads to the weakening of the ^5^D_3_ to ^7^F_J_ transition, thus leading to the strengthening of the ^5^D_4_ to ^7^F_J_ transition [122]. Various reported nanophosphors with doping of Tb^3+^ possessing afterglow emission falling in the wavelength region of green are: Lu_2_O_3_ doped with Tb^3+^ [198], CaSnSiO_5_ doped with Tb^3+^ [188], CaZnGe_2_O_6_ doped with Tb^3+^ [56], Y_2_O_2_S: Tb^3+^ co-doped with Sr^2+^, Zr^4+^ [199] etc. Another activator ion generally incorporated in luminescent materials for white emission is Dy^3+^. Its white emission is caused by transitions such as ^4^F_9/2_ to ^6^H_15/2_ and ^4^F_9/2_ to ^6^H_13/2_ of the Dy^3+^ ion. The main procedure for generating white light is by mixing red, green, and blue emission, also called RGB emission, but this method suffers some disadvantages, such as the fact that it does not attain optimized CRI and CCT values, and another limitation is that it is very difficult to achieve the same duration of afterglow for red, green, and blue PerLum phosphors. This activator ion possesses a huge potential for the application of white light afterglow emission. Two important emissions generated by Dy^3+^ ions lie in the wavelength range of 470–500 nm (^4^F_9/2_ to ^6^H_15/2_), corresponds to blue emission, and 570–600 nm (^4^F_9/2_ to ^6^H_13/2_), corresponds to yellow emission. Some other examples of PerLum nanomaterials with white afterglow resulting from doping with Dy^3+^ are Ca_3_SnSi_2_O_9_ [200] Sr_2_Al_2_SiO_7_ [201]^,^ CdSiO_3_ [121], CaMgSi_2_O_6_ [202], CaSnSiO_5_ [188], Sr_2_SiO_4_ [203,204] etc.

Many other RE-ions used as activators for PerLum are Tm^3+^, Yb^2+^, and Yb^3+^, which have been reported rarely as the centers of luminescence because of the difficulty in finding a suitable host. Su et al. [205], used Tm^3+^ as a dopant for Zn_2_P_2_O_7_ and observed a blue afterglow owing to transitions of the Tm ion, viz., ^1^D_2_ to ^3^H_6_, ^1^D_2_ to ^3^H_4_, and ^1^G_4_ to ^3^H_6_. They used UV light for excitation, and after it was switched, strong blue emission was obtained with an afterglow duration of over 1 h before its intensity decreased to less than 0.32 mcdm^−2^. Green afterglow has been observed with Sr_4_Al_14_O_25_: Yb^2+^ co-doped with Dy^3+^ [206] and SrAl_x_O_(1+1.5x)_: Yb^3+^ (x = 3,4,5) [206]. In the case of SrS doped with Yb^2+^, the afterglow obtained was found to be in the wavelength range of red, and was attributed to the 4f^13^5d^1^ - 4f^14^ transition of the Yb^3+^ ion [207]. Caratto et al. [208], synthesized a series of hexagonal II-type Gd oxycarbonate PerLum nanophosphors (Gd_2-x_Yb_x_O_2_CO_3_), doped them with Yb^3+^, and got an afterglow of approximately 144 h. They observed that these nanophosphors showed a very strong emission in the near IR region, and one major advantage of this nanophosphor was that its luminescence was observed to be independent of temperature, thus making this material a potential candidate to be used as an optical bio-label for bio-imaging.

#### 3.2.2 Transition and main group metal ions as activators

One of the most important transition metal ions used as an activator due to the 3 d^5^ electronic configuration, which features a broad emission starting from blue-green photons corresponding to 490 nm to the far-red wavelength corresponding to 750 nm, is Mn^2+^. This type of emission is attributed to the 3d–3d inter-atomic transition that is parity forbidden, going from the lowest excited state ^4^T_1_ to ^6^A_1_ ground state. The coordination number is also very important for the type of afterglow obtained in Mn^2+^-activated PerLum nanophosphors, as with Mn^2+^, tetrahedral coordination gives rise to green emission, while octahedral coordination results in orange to red emission [209,210]. For example, the afterglow of β-Zn_3 (_PO_4) 2_: Mn^2+^ co-doped with Ga^3+^ and γ-Zn_3_(PO_4_)_2_: Mn^2+^ co-doped with Ga^3+^ was observed to be very distinct, with the former displaying a strong red afterglow and the latter displaying a green and red afterglow. This property was attributed to two different coordination numbers, with β-Zn_3_(PO_4_)_2_:Mn^2+^, Ga^3+^ having a coordination number of 6 and γ-Zn_3_(PO_4_)_2_:Mn^2+^, Ga^3+^ possessing a coordination number of 4 as well as 6 [211]. Because of similar reasons, Mn^2+^-doped Zn_2_GeO_4_ displays a green afterglow [212]^,^ while Mn^2+^-doped compounds such as Li_2_ZnGeO_4_ [213], CdSiO_3_ [214], CaZnGe_2_O_6_ [215], MgGeO_3_ [216] display an afterglow emission in the orange-red region. Phosphorescence by Mn^2+^-activated compounds also includes Mn^2+^-doped glasses and nitrides, *e.g.,* AlN doped with Mn^2+^ [217]. Mn^4+^ is also a very important activator ion because of its 3 d^3^ electronic configuration, which ensures the stabilized emission of near-IR photons in the wavelength range of 600–800 nm in a variety of hosts. This emission has been observed to depend on the strength of the crystal field environment of the host used. Li et al. [218], synthesized Mn^4+^-doped LLPs, viz., MAlO_3_, where M denotes La and Gd, and obtained a peak emission at 730 nm with an afterglow of over 20 h. Pan et al. [219], synthesized a PerLum nanophosphor Zn_1+x_Ga_2-2x_Sn_x_O_4_:Cr^3+^ with emission in the near IR range that could be activated using low energy photons and recharged with an efficiency 400 times as compared with ZnGa_2_O_4_ doped with Cr^3+^ (used as reference material). They observed that by tuning the field strength of the crystal and the energy of the band gap with the help of cation occupancy, red-shifted PerLum could be obtained. This shift in luminescence properties made it a potential candidate for deep tissue imaging with better efficiency.

Among transition metals, another very important ion used as an activator is Cr^3+^ because of its 3 d^3^ electronic state, which ensures a narrow emission band at 700 nm. This emission is attributed to the ^2^E to ^4^A_2_ transition of Cr^3+^, which is spin forbidden. This activator ion also features a broad emission in the wavelength range of 650–1000 nm that is spin-allowed and attributed to the ^4^T_2_ to ^4^A_2_ transition of Cr^3+^. PerLum from nanophosphors doped with Cr^3+^ was for the first time observed by Bessiere et al. [220], in the year 2011, who detected an afterglow displaying a peak at 695 nm inZnGa_2_O_4_ crystals. In the year 2012, Pan et al. [133], synthesized a novel host, Zn_3_Ga_2_Ge_2_O_10_, and doped it with Cr^3+^, thus achieving an afterglow of 360 h. Li et al., used a technique of partial substitution of zinc and tin in place of gallium and thus synthesized the nanophosphor Zn_3_Ga_2_SnO_8_. After doping this nanophosphor with 0.5 % of Cr^3+^, they were able to achieve a strong afterglow that lasted for 300 h in the wavelength range of near IR, thus allowing imaging of deep tissues with the advantages of being extra-long, real-time, and reliable. Most of the hosts where the activator ion used is Cr^3+^ are gallate-based nanophosphors, which allow the emission of photons in the visible and near-IR ranges. Afterglow in gallate-based compounds activated by Cr^3+^ ranges from tens of seconds [221] up to a maximum of 1000 h [222]. Some examples of such gallate-based PerLum nanomaterials are La_3_Ga_5_GeO_14_:Cr^3+^ with an afterglow of 8 h [223,224], Zn_3_Ga_2_Ge_2_O_10_: Cr^3+^ with an afterglow of 360 h [133,225], LiGaO_5_:Cr^3+^ with an afterglow of 1000 h [222,226,227], Ca_3_Ga_2_Ge_3_O_12_:Cr^3+^ co-doped with Yb^3+^ and Tm^3+^ with an afterglow of 7000 s [228,229]. In case of these gallate-based nanophosphors, Cr^3+^ ions replace Ga^3+^ ions with a coordination of 8. After gallate-based LLPs, Cr^3+^ can also be used as an activator in other non-gallate nanophosphors, for example, Zn_2_SnO_4_:Cr^3+^ and Zn_(2-x)_Al_2x_Sn_(1-x)_O_4_:Cr^3+^, which show a broad emission spectrum ranging from 650 to 1200 nm with a peak at 800 nm and an afterglow duration of over 35 h [230].

Some of the main group elements have also been used as emission centers, and among them, Bi^3+^ is used as an activator for the generation of white light with light-emitting diodes. Luminescence, because of this activator ion, also depends on the coordination number of emission centers, which ensures luminescence ranging from blue to red emission. Some of the Bi^3+^-activated PerLum materials include CaWO_4_ [231,232], ZnGa_2_O_4_ [233], CdSiO_3_ [56,234] and Zn_2_GeO_4_ [235]. In case of all these nanophosphors, the afterglow observed was found to be in the wavelength range of UV-blue region. Also, their pattern of luminescence is influenced both by Bi^2+^ and Bi^3+^, thus giving rise to visible or near-IR PerLum.

## 4 Synthesis of persistent luminescence materials

Materials which show the phenomenon of PerLum are inorganic materials with high purity synthesized via different methods. A phosphor mainly comprises of a host material such as silicate, aluminate, phosphate, oxide, stannate, sulphide, titanate, nitride, halide, etc., and activator ions [209,[236], [237], [238], [239], [240], [241]]. To improve the LLPs of such phosphors, a sensitizer is also added, which acts as a mediator of energy transfer between the excitation source and activator ion [242]. For better efficiency and improved properties, phosphors must possess properties such as extra-small size, a large surface-to-volume ratio, better morphology, and a narrow particle size distribution. The method employed for the synthesis of LLPs has a very important role in determining their properties, efficiency, time of afterglow, morphology, and yield [19]. Different synthesis methods employed have their own advantages and disadvantages, which shows that it is very important to select an appropriate method of synthesis. With the solid-state method of synthesis, the strength and duration of the afterglow are improved, but the morphology and dispersibility of synthesized LLP were found to be very poor [12,14]. While combustion synthesis is advantageous in the sense that it is quick, sample, needs low energy input and generates high purity sample it also has a disadvantage of product being agglomerated along with lack of control over the morphology of the final product and shorter duration of afterglow. Other wet-chemical methods, which do not need high annealing temperatures, result in the synthesis of small molecules with improved mono-dispersity but weak emission and a shorter afterglow. Table 1 provides details about recently synthesized phosphors, along with their various characteristic properties and applications. Also, a brief description about different synthesis methods along with their merits, and demerits is discussed below.

**Table 1:** Details related to some recent PerLum materials along with different dopants and co-dopants used, afterglow exhibited by them, emission wavelengths and their applications showing their importance in modern technologies and day-to-day life.

Synthesis technique	S. No.	Host employed	Dopant/co-dopant	Afterglow duration	Emission wavelength	Application	Ref.
Solid state synthesis technique	1.	ZrO_2_	Tb^3+^	600 s		Radiation dosimeter in biomedical for excitation using beta rays	[243]
	2.	(Ba,Sr)Ga_2_O_4_	Sm^3+^	1400 s	500 nm–750 nm	Multimodal material for anti-counterfeiting	[244]
	3.	ZnGa_2_O_4_	Ni^2+^	More than 500 s	1300 nm	NIR–II Window, In-vivo imaging	[245]
	4.	Ba_2_MgSi_2_O_7_	Eu^2+^/R^3+^ (R=Y,La–Nd, Sm–Lu)	6 h	500 nm		[246]
	5.	NaGdGeO_4_	Bi^3+^/Li^+^	More than 200 h	325 nm–525 nm, peaking at 400 nm	Biomedical	[247]
	6.	BaGa_2_O_4_	Pr^3+^	3500 s	400 nm–550 nm	Anti-counterfeiting	[248]
Combustion synthesis	1.	(M,Ca)AlSiN_3_ (M = Sr, Mg)	Eu^3+^	200 min.	500 nm–750 nm	Security, display, data storage	[249]
	2.	AlN	Mn^2+^/Si^4+^	380 s–1200 s	600 nm	Lighting and display	[250]
	3.	Ca_2_Ga_6_O_14_	Pr^3+^	10.3 h	430 nm–510 nm	Data storage	[251]
	4.	Sr_2_MgSi_2_O_7_	Eu/Dy	5 h	456 nm	Field emission display	[252]
	5.	MAl_2_O_4_ (M = Sr, Ba, Ca)	Eu^2+^/R^3+^ (R=Dy, Nd, La)	7 h	516 nm–500 nm, 400 nm		[253]
	6.	CaAl_2_O_4_	Eu^2+^/Dy^3+^	6 h	438 nm	Displays, detectors, data storage	[254]
Hydrothermal method	1.	LiGa_2_O_8_	Cr^3+^	10 min.	425 nm, 610 nm	Bioimaging	[255]
	2.	Bi_2_Ga_4_O_9_	Cr	More than 30 min	696 nm, 706 nm	Computed tomography imaging, long term and sensitive diagnosis,	[256]
	3.	Zn_3_Ga_2_SnO_8_	Cr^3+^/Yb^3+^, Er^3+^	60 min	700 nm	Advanced imaging therapy, high resolution bioimaging	[257]
	4.	Zn_3_Ga_2_Ge_2_O_10_	Cr^3+^	15 h	697 nm	In vivo bioimaging	[258]
Co-precipitation method	1.	NaYF_4_	Ln^3+^	1800 s	480 nm–1060 nm	Optical data storage, luminescent inks	[259]
	2.	Sr_3_SiO_5_	Eu^2+^	7000 s	580 nm	White LEDs	[260]
	3.	BaMoO_4_	Tm^3+^	5.7 days	453 nm, 545 nm		[261]
	4.	Ca_3_(PO_4_)_2_	Mn^2+^, Ln^3+^ (Ln = Dy,Pr)	10 min	480 nm, 575 nm, 612 nm, 626 nm, 665 nm	In vivo imaging	[262]

### 4.1 The solid-state reaction method

**Fig. 8:**
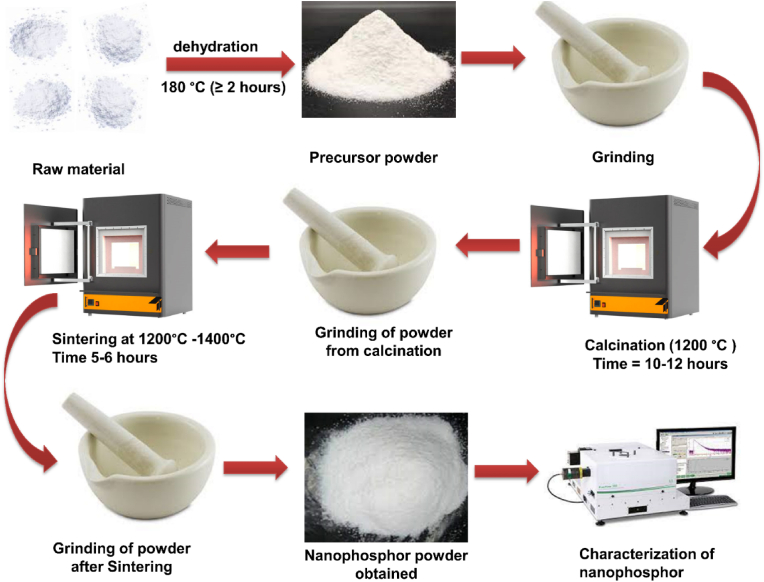
Diagram representing the general procedure of solid-state reaction for the synthesis of persistent luminescent nanomaterials.

For the synthesis of LLP, a solid-state synthesis technique is employed, as this method ensures a longer duration of afterglow and strong luminescence. With a solid-state reaction, the physiochemical property of the synthesized material gets changed. Some of the primary factors that alter the rate of reaction are temperature and other physical conditions, reactivity, and the structural and surface properties of reactants [263]. The general procedure of solid-state reaction is shown in Fig. 8, from which we can understand the different steps involved in the process of synthesis of nanophosphors via the solid-state reaction method. In this method of synthesis, all precursors are weighted and then mixed while staring at low temperatures [264]. For decreasing the temperature needed, improving the crystallinity, and increase the rate of reaction, different fluxes such as Li_2_CO_3_, YF_3_, AlF_3_, NH_4_F, BAF_2_, boric acid, etc., are used. Apart from these, the addition of flux also helps in enhancing the temperature of the reaction must be very high because, at higher temperatures, reactants react at a faster rate, which ensures lower activation energy. The temperature during a solid-state reaction varies in a range of 1200–1600 °C, which generates a product in powder form. Other advantage of this synthesis method is that no toxic gases or waste are generated during this procedure. However, this method suffers from disadvantages such as the need for higher temperatures, a longer duration of time, weak surface morphology, low dispersibility, and homogeneity [265]. With solid-state reactions, the yield is quite good, which makes it an ideal technique for large-scale production. This technique has been most widely used for the synthesis of PerLum nanophosphors because of its already discussed advantages [[265], [266], [267], [268]]. While discussing the mechanism of LLP, as already mentioned, traps and emitters are of primary importance for this phenomenon, and high temperatures have proved very helpful for inducing lattice defects, distortions, vacancies, intrinsic defects, etc. In 2011, Pan and others [269] synthesized sunlight-activated near-IR PerLum nanophosphor via solid-state reaction. They observed that these zinc-gallogerminate ceramic discs, doped with Cr^3+^, displayed a very long afterglow of over 360 h. Different measurements showed that high temperatures induced the formation of oxygen and gallium vacancies, due to which trapping states with very high densities were formed. Same group also synthesized germanite-based nanophosphor LiGa_2_O_8_ doped with Cr^3+^ with near IR photo-stimulated LLP. This nanophosphor showed a much longer afterglow of 1000 h and was used for optical information storage [226]. Cai et al.*,* [270] employed both high temperature solid state technique and hydrothermal technique to synthesize Cr^3+^ Zn_1.33_Ga_1.34_Sn_0·33_O_4_. The authors have presented that further grinding of samples can significantly impact the properties of PerLum, resulting in a reduction in intensity.

### 4.2 Solution combustion method

**Fig. 9:**
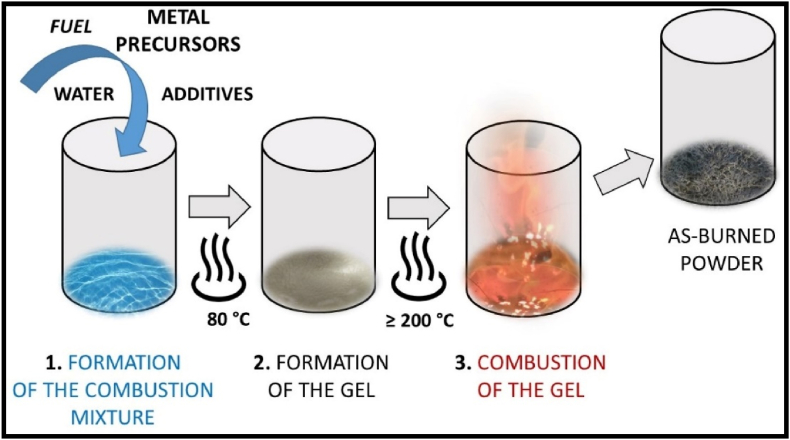
Representation of the step-by-step process of solution combustion synthesis. Reproduced with permission from Ref. [283], Copyright 2017, Elsevier.

The solution combustion method is a rather simple and easy method for the synthesis of nanophosphors. This method has the potential to save energy and time, and it can synthesize several phosphors, such as oxides, aluminates, sulphides, and highly reactive alloys [[271], [272], [273], [274], [275], [276]]. During this synthesis method, nitrates of different metals are mixed along with a suitable fuel which acts as a reducing agent, such as sucrose-PEG [277], urea-PVA [278], hexamine-PVA [278], urea-starch [279], cellulose-citric acid [271] *etc*. . Reducing the power of fuel is of primary importance for its selection because higher the reducing power, better will be the fuel. For this reason, urea or citric acid is used as fuel because of their high reducing power, low cost, and ease of availability. During this method of synthesis, heat is generated as it is exothermic, which results in high crystallinity in the synthesized materials. Phosphors synthesized via this method are Lu_3_Al_5-x_Ga_x_O_12_ doped with Ce^3+^ and co-doped with Cr^3+^ [280], Y_3_Al_5_O_12_ doped with Pr^3+^ [281], AlN doped with Mn^2+^ and co-doped with Si^4+^ [250], Gd_3_Al_2_Ga_3_O_12_ doped with Ce^3+^ and co-doped with Cr^3+^ [282]. Synthesis of phosphors via this method primarily includes four steps (Fig. 9 [283]), viz. (a) preparation of a mixture of precursors and a suitable fuel such as urea or citric acid (b) preparation of gel by mixing them with distilled water and continuously stirring at an approximate temperature of 80–100 °C (c) Combustion of gel with the help of a furnace set at a temperature greater than 500 °C. Combustion of gel generates powder with a sub-micron size, which is crushed into fine powder. (d) This powder is then calcinated at a higher temperature to remove some extra impurities and to get better crystallinity and improved luminescence, which results in the generation of powder with high purity [273,284,285]. Some main advantages of the combustion method are better morphology, better dispersibility, easy processing, and saving energy and time, but while synthesizing PerLum materials, there are some disadvantages as well, such as a small afterglow time and a weak emission intensity.

### 4.3 Template method

Synthesis of PerLum nanoparticles via the solid-state method generates LLP with longer afterglow and strong emission, but one of the main limitations of this method is that synthesized nanophosphors have weak morphology, irregular shape, and poor homogeneity because of the high annealing temperature, due to which such LLP cannot be used for biological applications [[286], [287], [288], [289]]. In contrast to this, wet synthesis methods generate nanophosphors with better morphology and regular shape, which are ideal for biomedical applications. The template method is one of the well-established processes for the synthesis of near-IR PerLum nanophosphors. In this method, metal ions, which act as precursors, are first infused into the mesopores of templates. The second step during this procedure is the annealing of the prepared sample to get uniform size and morphology, along with a fine size distribution [14]. With the help of this method of synthesis, Zhang and his co-workers synthesized many PerLum nanophosphors, such as mSiO_2_@Gd_3_Ga_5_O_12_ doped with Cr^3+^ and co-doped with Nd^3+^ [290], ZGOCS@MSNs@Gd_2_O_3_ [291], ZnGa_2_O_4_ @HMS [292], etc. Near-IR PerLum nanomaterials synthesized using this method possess features of mesoporous structure, uniform distribution, and spherical morphology. The structural morphology and dimensions of nanophosphors can be varied by varying the contents of precursor mesoporous silica nanostructures. Zhang et al. [293], synthesized a near-IR PerLum nanomaterial with a hollow structure and used this material for the imaging of tumors and chemo-photodynamic synergistic therapies. Because of its hollow structure and large size after calcination, this synthesized nanomaterial allowed the loading of an extra amount of drugs and photosensitizer. It was found that the template method for synthesis of near IR PerLum nanomaterials with a hollow structure has huge potential for biomedical applications [294].

### 4.4 Hydrothermal method

For the synthesis of near-IR PerLum nanophosphors, the template method has proved very helpful in terms of improved morphology and homogeneity, but due to the need for high temperatures during calcination, some important functional groups are lost, and the synthesized near-IR PerLum nanophosphor may have limited function when it is used for biomedical applications [13]. For improvement of biocompatibility and surface functionalization, another method is employed for the synthesis of LLPs called the hydrothermal or solvothermal method [295]. This method of synthesis uses a rather different approach, where synthesis is done by growing a crystal inside an aqueous solution kept at a higher temperature or an organic solution kept at a high pressure and temperature (less than 300 °C) [296]. This method of synthesis has many advantages, such as the fact that it can help in the synthesis of nanophosphors with a controlled size of particles, improved morphology, and a monodisperse product. With a change in the reaction's temperature, pH of the medium, time of reaction, and concentration of precursors, the properties of the product can be changed [297]. Han et al. [298], used this method to synthesize near-IR PerLum nanophosphors, in which they employed the method of direct aqueous phase reaction to synthesize near-IR PerLum nanostructures having a size under 10 nm. Such nanoparticles having a size under 10 nm allowed efficient surface functionalization and could easily form aqueous colloidal solutions and cell culture medium, so they could be used in biomedical applications. With the help of hydrothermal synthesis techniques, the development of near-IR PerLum nanomaterials, which have many potential applications in medicine and bio-imaging, has become possible. Because of this reason, such materials are called “luminous pearls” and are very important for biomedical applications [299,300]. Mao and coworkers [301] employed a dual-phase hydrothermal procedure to synthesize ZnGa_2_O_4_ doped with Cr^3+^ with near-IR PerLum. With the help of the hydrolysis of precursors in an aqueous solution of water and toluene, they could control the size of the synthesized nanomaterial. The pH value of the medium is also very important because an alkaline pH can cause the formation of monodisperse nanoparticles with a size of less than 10 nm [13]. Zhau et al. [302], proceeded via a modified hydrothermal method where they used oleic acid as a surfactant, and during this procedure known as the solvothermal liquid-solid solution (LSS), they changed the temperature of the reaction and molar ratio of Zn and Ga precursor ions and could synthesize LED-activated, rechargeable near-IR PerLum monodisperse nanomaterials. During this process, they were able to control the size of nanoparticles up to 4–10 nm, and because of their smaller size, such PerLum nanophosphors possess a high potential for modification of functional groups as well as bioconjugation. Since oleic acid has been used as a surfactant, which increases the duration of afterglow along with increasing the strength of emission.

### 4.5 Sol-gel method

**Fig. 10:**
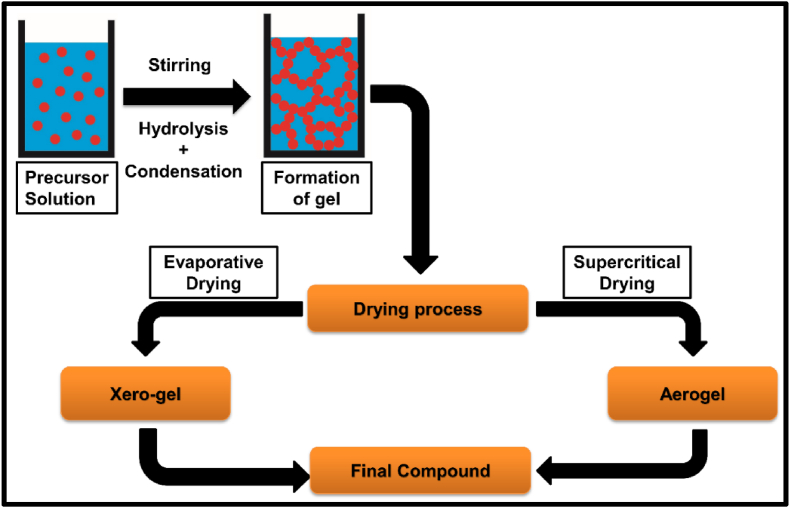
Schematic representation of the sol-gel method of synthesis for metal oxide nanostructures which involves the formation of gel via constant stirring thus allowing hydrolysis and condensation that results in the formation of the gel.

For the synthesis of nanophosphors with strong emission, this method has proven very effective while being efficient and cost-effective at the same time [303,304]. This process has received a lot of attention for the synthesis of inorganic PerLum nanophosphors. This process involves the use of precursors of metal ion salts or alkali oxides. Citric acid, used as one of the key ingredients during this process of synthesis, acts as a chelating ligand, and alcohol is used as a cross-linking agent for the formation of polymeric resin. Owing to the requirement of heat treatment in the sol-gel method, which is very important for the PerLum property, this method is often used for preparing LLPs. Aluminate and silicate-based LLP have been widely synthesized via this route of synthesis [118,305]. Near IR PerLum nanophosphor synthesized via this technique by Chermont et al. [33], is Ca_0·2_Zn_0·9_Mg_0·9_Si_2_O_6_:Eu^2+^ co-doped with Dy^3+^ and Mn^2+^. They reported the use of this nanophosphor for the application of bioimaging. The analysis of the sample revealed that the size of the nanoparticles was as small as 50–200 nm. The sol-gel method has become very popular, and many other researchers have also employed this method for the synthesis of nanomaterials, e.g., Zn_3_Ga_2_Ge_3_O_10_ doped with Cr and co-doped with Pr [306], ZnSiO_4_ doped with Mn [307], CaMgSi_2_O_6_ doped with Mn [308]^,^ etc. Apart from the smaller size, the sol-gel method of synthesis also helps in controlling the morphology of the final product by varying the synthesis parameters of the reaction [309,310]. Fu et al. [311], synthesized LiGa_5_O_8_ via the sol-gel mathed. During their study, they studied the effect of temperature on the size distribution and morphology of the final product. They found that the size of nanoparticles varies from 50 to 300 nm when the temperature goes from 700 to 1100 °C. This is a very simple technique used to synthesize silica-based nanophosphors with strong emissions. With the help of this technique, nanomaterials with lattice structures such as those of sulphides, borates, phosphates, halides, *etc.*, have been synthesized [312]. In the year 2020, Kim et al. [280], synthesized Lu_3_Al_5-x_Ga_x_O_12_: Ce^3+^, co-doped it with Cr^3+^ via the sol-gel combustion method, and studied its PerLum properties. After studying its photoluminescence spectroscopy, they found that it was comprised of strong bands of Ce^3+^ while having weak bands of Cr^3+^ in the wavelength range of the green and red regions, respectively. Lu_3_Al_3_Ga_2_O_12_: Ce^3+^ co-doped with Cr^3+^, which was synthesized at 1700 °C and an afterglow of 40 min was obtained [280]. In the year 2014, Teng et al. [313], studied PerLum of strontium aluminate doped with Eu^2+^ and co-doped with Dy^3+^ and Cr^3+^ synthesized with the help of the sol-gel combustion synthesis method. The luminescence from this nanophosphor had a peak at a wavelength of 760 nm. They reported that PerLum from Cr^3+^ lasted for a couple of hundred seconds, while introducing Dy^3+^ extended PerLum in this nanophosphor was obtained. In the year 2021, Ge et al. [314], used the sol-gel method to synthesize ZnGa_2_O_4_ doped with Cr^3+^ and got an afterglow of 2 h with an emission wavelength of near IR. They proposed that this PerLum nanophosphor would provide bright and efficient imaging. With the help of this procedure, many other phosphors, such as M_2_SiO_4_ doped with Tb^3+^ and Y_2_Si_2_O_7_ doped with Eu^3+^, were also synthesized [315]. Fig. 10 represents the general process of synthesis of nanomaterials via the sol-gel method [316].

### 4.6 Coprecipitation method

As the name suggests, this method involves the precipitation of substances that are soluble in normal conditions. In this method, coprecipitation is done by dissolving raw materials in some solution and then, with the help of some reagent cations is being co-precipitated. These cations are then separated, and, with the help of high temperatures, these precursors are decomposed, and finally, with the help of annealing, powder is crystallized. Because of efficient mixing, this technique is used for the synthesis of inorganic nanomaterials, as it provides improved interspersion and thus results in the formation of a more homogenous product. Nowadays, this technique is also being used for the synthesis of fluorides and oxides, which show luminescent properties. As already discussed, the properties of PerLum require high temperatures. Thus, in the case of co-precipitation, a simple modification of high-temperature treatment can be done so that this method can be used for the synthesis of LLPs, for example (Y_1-x_Gd_x_)_2_O_3_:Eu^3+^ and co-doped with Sm^3+^, Si^4+^, and Mg^2+^ [317]. Cheng and others synthesized strontium aluminate doped with Eu^2+^, co-doped with Dy^3+^, and barium aluminate doped with Eu^2+^ and co-doped with Dy^3+^ nanotubes with a hexagonal structure that displayed the property of PerLum. For an efficient PerLum, the final product was given heat treatment along with a reducing atmosphere. After analyzing these nanotubes under a transmission electron microscope, it was found that they possessed a wall thickness of 30–60 nm and were approximately 80–200 nm in diameter. But these nanotubes suffered a limitation in that they couldn't be used as bio-labels for optical imaging [318,319].

## 5 Properties of LLPs

Characteristics of LLPs differ from fluorescent materials in the way that once excited, they store energy in traps and defects, and then when excitation stops, controlled emission of energy starts at room temperature due to de-excitation of charge carriers. As already mentioned, this luminescence lasts for a duration of a few seconds to a couple of hours [8,58]. Emission and excitation characteristics of PerLum nanophosphors depend on many factors, such as the type of traps, their spatial distribution, the depth of traps, and the excitation wavelength used. In this part of the review, we will discuss properties of the emission spectrum, excitation spectrum, different factors related to traps, and how they help in the enhancement of afterglow.

### 5.1 Excitation and emission characteristics

Excitation of LLPs is mostly done with the help of UV or visible light, but sometimes the use of ionizing radiation, such as X-rays, *etc.,* can be done for PerLum nanomaterials with a lower value of bandgap energy. Research has found that the use of optical excitation and ionizing radiation gives rise to the filling of different traps [320,321]. This phenomenon has a simple explanation: optical charging takes place via the direct excitation of an activator, while charging via ionizing radiation is assisted by the avalanche effect generated by charge carriers. For the estimation of the wavelength used for charging LLP, excitation characteristics are taken into consideration [322,323]. Research has shown that some LLPs can be excited with the help of photons with low frequency (e.g., for NIR PerLum, use of red light), but most of them can only be excited with the help of photons with higher energy, such as UV. Clercq et al. [324], synthesized NIR-emitting LLP LiGa_5_O_8_:Cr^3+^ in 2017 and attempted to excite it with the help of the red, blue, and UV energy spectrums, observing that only UV light could trigger PerLum. Maldiney et al. [29], observed the NIR-emitting PerLum nanophosphor ZnGa_2_O_4_:Cr^3+^ and concluded that it could be charged with the help of orange light, but efficient excitation takes place by using UV photons. The emission characteristics of a nanophosphor can be understood as the effect of temperature on the emission of a nanophosphor. After the discovery of PerLum, most of the attention was given to phosphors with emission in the visible range of the electromagnetic spectrum, as demanded by most of the applications.

**Fig. 11:**
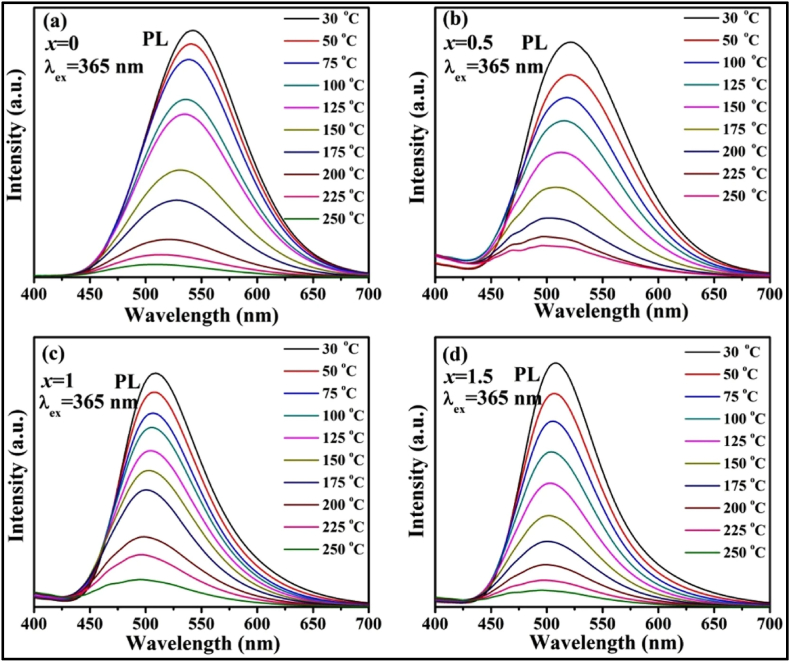
Results obtained from PL spectroscopy which represent the dependence of emission spectrum from Ca_2·97_Si_3-x_O_3+x_N_4-2x_:0.03Eu^2+^ (x = 0,0.05,1,1.5) excited by UV photons of wavelength 365 nm on temperature. Reproduced with permission from Ref. [325], Copyright 2017, Springer Nature.

In the year 2017, Wang et al. [325], synthesized a series of Ca_3_Si_3-x_O_3+x_N_4-2x_:3 % Eu^2+^ (x = 0, 0.2, 0.3, 0.4, 0.5, 0.6, 0.8, 0.9, 1, 1.2, 1.4, and 1.5) nanophosphor via high-temperature solid-state reaction and studied the effect of temperature on emission spectrum by taking x equal to 0, 0.5, 1, and 1.5 as shown in Fig. 11 [325]. For nanophosphors to have visible emission dopants, mostlyEu^2+^ is used, while varying the host can give rise to tuning of emission from violet to deep red [57]. In recent years, many applications such as bio-imaging, latent fingerprinting, and anti-counterfeiting have demanded the emission from PerLum nanophosphors to be between the UV [[326], [327], [328]] or infrared spectrum [226,329,330]. As emission from Eu^2+^-doped nanophosphors lies in the visible energy spectrum, for UV or visible emission, different dopants such as Pr^3+^, Pb^2+^, Bi^3+^, or Ce^3+^ were used. Phosphors with afterglow emission in the wavelength range of NIR have proved very beneficial for applications such as drug delivery, bio-imaging, etc. For such applications, the most commonly used dopants are Cr^3+^ [220,226,324], Mn^2+^ [216], Mn^4+^ [331,332] *etc.*

### 5.2 Properties of traps

**Fig. 12:**
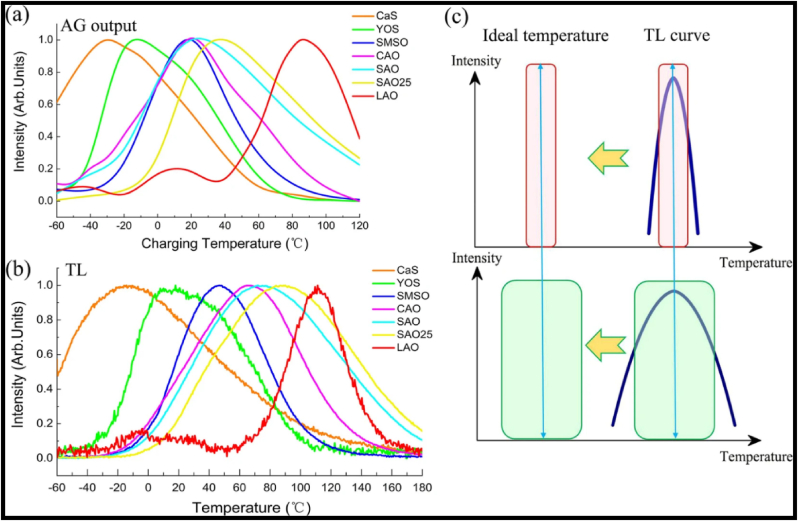
(a) Intensity vs temperature graph representing comparison between normalized afterglow of different Per Lum nanophosphors, (b) Intensity vs temperature graph representing thermoluminescence glow curves of different Per Lum nanophosphors, (c) Schematic representation showing relation between optimum temperature and thermoluminescence glow curves. Reproduced with permission from Ref. [333], copyright 2019, Springer Nature.

PerLum assisted with temperature shows a large dependence on the distribution and depth of the trap, and the efficiency and application of PerLum depend on the temperature of the surroundings. Thus, to estimate the performance of nanophosphor, it is very important to take the optimum temperature into consideration, which is the temperature at the LLP that shows maximum afterglow. The optimum temperature for a nanophosphor is decided with the help of the highest peak attained during thermoluminescence measurement, as this temperature is about 20–30 °C below the peak glow temperature To understand the effect of temperature on the properties and applications of luminescence, we can consider an example of CaS:Eu co-doped with Dy and SrAl_2_O_4_:Eu co-doped with Dy. CaS shows red luminescence, and its thermoluminescence studies show peak emission at −20 °C, so this nanophosphor is considered better for low-temperature operations, while thermoluminescence studies of SrAl_2_O_4_ show that its use is well suited for applications operating at room temperature. Fig. 12 (a, b) [333] show the dependence of luminescence on temperature for different nanophosphors with the property of PerLum. It has been observed that the shape of peaks regarding temperature and the location of peaks are the same as predicted by thermoluminescence glow curves, as shown in Fig. 12 (c) [333]. PerLum nanomaterials with shallow traps release charge carriers quickly, and thus the optimum temperature is very low as compared to room temperature, while deep traps do not release charge carriers at lower temperatures, thus showing PerLum at higher temperatures [334]. Thus, it is very important to generate traps with specific depths so that the start of PerLum at a certain temperature is ensured. Analysis of a variety of PerLum materials has shown that they possess different trap depth distributions rather than having a well-defined trap depth, which proves different trapping defects. The relation of different defects with particular trap depths is not very well known, but some explanations for different trap depths and different values of optimum temperatures given over time are due to addition of co-dopants and variation in their chemical environment, the distribution of traps, and the properties of the recombination center. To understand PerLum and its relation to trap depths, different compounds were studied theoretically, e.g., the study of ZnGa_2_O_4_:Cr^3+^ for anti-site defects [335,336]. With the help of X-ray spectroscopy, it has been observed that with Sr_4_Al_14_O_25_: Eu co-doped with Dy, trapping takes place when Dy^3+^, after absorbing, gets converted into Dy^2+^ [337]. The relationship between trap depths and different defects is still a topic that needs a lot of research for better understanding. To evaluate the efficiency of LLP, variation in intensity of afterglow regarding time is recorded after a PerLum nanomaterial is excited by a standard excitation source [338]. Units to express the afterglow of a nanophosphor are expressed according to the type of emission. For example, the afterglow of LLPs having an emission wavelength in the visible region of the electromagnetic spectrum is often expressed in terms of photometric units, while the afterglow of luminescent materials with an emission wavelength in the IR region of the electromagnetic spectrum is expressed in terms of radiometric units. Thus, comparing the afterglow curves of different curves is rather difficult, as the afterglow of some nanophosphors is expressed in arbitrary units as well.

**Fig. 13:**
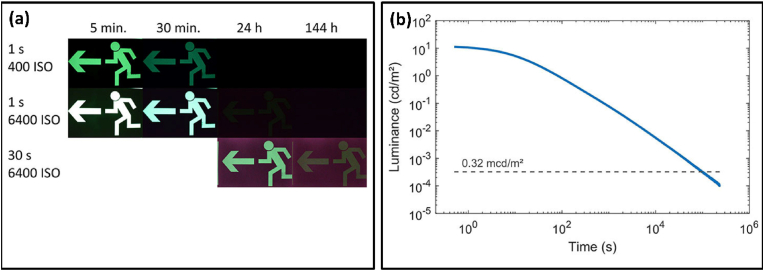
(a) Photographic image of a commercially used exit sign which glows in dark. These photographs have been taken at different times after the end of the excitation along with different camera settings. The authors have used a Nikon D3200 digital camera with an objective lens having a focal length of 35 mm and a numerical aperture of f1.8. they used JPG images without any processing and the excitation source used was a blacklight lamp at 365 nm for 10 min. (b) Decay curve showing the variation of intensity of afterglow of the same exit sign where the benchmark intensity of 0.32 mcd/m^2^ is reached after 27.5 h. Reproduced with permission from Ref. [42], Copyright 2020, American Institute of Physics.

The morphology of LLP also plays a very important role in its properties, with its morphology ranging from transparent glass-ceramic types displaying somewhat enhanced afterglow to powder forms with sizes in the order of 10^−9^ m. For comparing the intensity of different nanophosphors and their variation over time, it should be expressed in terms of cd/m^2^, and thus most of the data can be gotten from afterglow curves, which are measured over a longer duration of time. To show a change in the intensity or strength of afterglow photographic images along with time, after excitation are recorded, thus expressing it in terms of its visibility to the naked human eye, as shown in Fig. 13 (a). To determine the time of afterglow, the benchmark value of intensity used is 0.32 mcd/m^2^, which is the value of intensity that can be detected by a human eye adapted to a dark environment. Fig. 13 (b) [42] represents the same benchmark, where it can be observed that the benchmark of 0.32 mcd/m^2^ is achieved after 27.5 h from the end of excitation. Using photometric units for comparison of afterglow rarely generates good results. This is because of an effect known as the Purkinje effect, which states that the sensitivity of the human eye shifts not only to shorter wavelengths with a decrease in the intensity of light but also because the eye adapts to a dark environment [339]. The shift of vision from photopic to scotopic can be described as a complex process that does not permit the differentiation between the physical appearance of the source of light under different conditions, but in some simple cases it is possible to differentiate, which has thus led to the concepts of unified luminescence [340] and visibility index [338,341,342]. Now if PerLum in the wavelength range of NIR or UV is taken into consideration, then it is imperative to use a radiometric unit, i.e., mW/m^2^/sr to compare the afterglow of different such LLPs, as by definition their afterglow is zero in terms of cd/m^2^ [52]. The luminous efficacy of radiation, denoted by K, is a measure of eye sensitivity in a particular part of the electromagnetic spectrum. The value of K for a nanophosphor emitting pure green light in the wavelength range of 555 nm is 683 lm/W, and for a nanophosphor such as SrAl_2_O_4_:Eu co-doped with Dy, the green emission value of K is approximately 450 lm/W. Since we know that 1 cd equals 1 lm/sr, it is possible to convert the benchmark of minimum detectable intensity with the naked human eye expressed in photometric units, i.e., 0.32 mcd/m^2^, into radiometric units, as 0.32 mcd/m^2^ equals 0.32/KmW/m^2^/sr. With the help of such a benchmark in terms of radiometric units, it is possible to compare the afterglow of LLPs with UV or NIR emission. If the average value of luminous efficacy for visible emission is expressed in terms of radiometric units, then the value of benchmark luminous efficacy for UV or NIR emission would be equal to 1 × 10^−3^ mW/m^2^/sr [343]. One more method to measure the efficiency of a nanophosphor is to measure its storage capacity, which is measured in terms of the number of photons emitted per gram of luminescent material after the excitation source is switched off. One major advantage of this method is that it allows comparison regardless of morphology and spectral characteristics. The benchmark for this comparison is SrAl_2_O_4_:Eu co-doped with Dy, which has been found to emit (1.57 ± 0.03) × 10^17^ photons per gram, which is equivalent to 1.6 % of Eu ions being ionized. This observation shows that there is still room for a lot of improvement, particularly when research in this field has shown that for other LLPs, storage capacity is much lower in magnitude [344]. Different steps involved in the measurement of storage capacity via measuring the number of photons have been explained by Van der Heggen et al., [344]. Another factor on which the performance of a nanophosphor depends is the type of excitation source used; e.g., most nanophosphors can be easily excited with the help of UV light as it fills the traps more quickly than photons with a longer wavelength because of the presence of thermal barriers during trapping, but sometimes it is very important to use photons with a specific wavelength, such as nanophosphors used in bio-imaging. Orange to red light is often used for excitation rather than UV photons [29].

## 6 Applications

The phenomenon of PerLum has been used for many applications over time because of its peculiar property of extended afterglow. These materials emit radiation over longer durations of time after excitation, which makes them an important part of bioimaging for the diagnosis of cancer and its guided treatment via the use of guided drug delivery, guided photo-thermal therapy, and photodynamic therapy. Using these interesting materials has also seen a boom in the biosensing of proteins and the detection of other small molecules. One of the very important applications of these material is in-vivo bio-imaging because it possesses all the required and expected properties, such as a size less than 100 nm, strong emission in the wavelength range of deep red to NIR corresponding to tissue transparency, a longer afterglow, the property of functionalization, and in-vivo stability [[345], [346], [347], [348], [349]]. Recent research has found the use of LLPs for the reduction of flickering in AC LEDs, as it has been found that PerLum nanomaterials can act as a bridge between dark periods in AC LEDs when they are not emitting. LLPs have also found their use in commercial safety markings and glow in dark toys and other objects. Such materials can also become an important part of solar panels as they can act as efficient down converters that convert high-frequency photons to low-frequency photons, thus increasing the efficiency of solar cells [64,350,351]. These materials have also been used as photocatalysts, wherein upon end-of-emission, LLP activates the photocatalyst, thus resulting in extended photocatalysis [352]. Some of the major and most important applications of PerLum have been explained in this review.

### 6.1 Biomedical applications of long-lasting phosphors

One of the major applications of PerLum nanoparticles (PLNPs) is in medicine and health care, and the main reason for this is the longer duration of the afterglow, which helps in avoiding any chances of autofluorescence and scattering of light because of various biological fluids, tissues, etc. Due to their extremely long afterglow and strong emission in the deep red to NIR wavelength range, PLNPs have been very promising for the applications of biosensing [[353], [354], [355], [356]], bioimaging [33,145,287,298,[357], [358], [359], [360], [361], [362], [363]] and theranostics [18,[364], [365], [366], [367], [368], [369], [370], [371], [372]]. Nanophosphors synthesized via high-temperature solid-state reactions have not been suitable for the biomedical field because of their irregular morphology and poor biocompatibility. Thus, nanomaterials with intended biomedical use must be synthesized via more traditional methods such as combustion, sol-gel, co-precipitation, etc., which allow controlled morphology at the nanoscale, better biocompatibility, and better dispersibility [19,373,374]. Nanophosphors for biomedical applications such as nano probes need to have some basic properties, such as an intense, long, yet stable afterglow with the property of renewability, and, size less than 200 nm for in vivo bio-imaging, the property of signal change triggered by a target for biosensing, targeting ability both active and passive, and multifunctionality for the applications of theranostics. Research in PLNPs has led researchers to a point where it has become possible to significantly enhance PL properties, produce a longer and more intense afterglow, and synthesize nanoparticles with control over size and morphology. Such advantages have led and promoted scientists to the biological use of such nanomaterials [14,19,262,373,374].

#### 6.1.1 Per Lum based bioimaging

Bioimaging based on fluorescent nanoparticles is a non-invasive, organ- or tissue-specific, and visual method used to detect and observe different biological processes. This imaging technique has proven to be an effective and efficient imaging technique in recent years [[375], [376], [377], [378]]. Bioimaging, with the help of fluorescent organic dyes, metal complexes, and quantum dots, possesses a need for excitation throughout the imaging process, which gives rise to autofluorescence in other biological molecules present in the vicinity [379]. The signals generated by autofluorescence interfere with the signals from the target, thus hampering the quality and accuracy of the image. Because the fluorescence lifetime of proteins and other small molecules is less than some nanoseconds, the use of PLNPs has been preferred because their luminescence lasts from a couple of minutes to several hours, due to which autofluorescence can be removed completely [33]. Thus, PerLum bio-imaging is considered an autofluorescence-free next-generation imaging technique. For the first time, this imaging technique was employed by Chermont et al. [33], in 2007 by using Ca_0·2_Zn_0·9_Mg_0·9_Si_2_O_6_:Eu^2+^ co-doped with Dy^3+^ and Mn^2+^ as a biomarker, thus laying the foundation stone for the use of PLNPs in the field of bio-imaging. A luminescent material that is used for bio-imaging should also possess the tendency to get degraded and afterward removed from the body to lower the cytotoxicity caused by it [380]. Lecuyar et al. [381], studied the degradation of ZnGa_2_O_4_:Cr^3+^ luminescent nanostructures within a medium resembling with a lysosome in 2020. They employed this nanomaterial for in vivo bio-imaging and studied the effect of long-term degradation on the in vitro toxicity of this nanophosphor. Lécuyer and colleagues [382] synthesized ZnGa_1.995_O_4_Cr_0.005_ nanoparticles that exhibit bright PerLum signal transmission through tissue with transparency window for several minutes. Additionally, these nanoparticles could be activated in-vivo through visible photons. Such distinctive properties make them suitable for long-term in vivo imaging studies without any background interference, enabling the recovery of valuable information.

Phosphorescent-based bio-imaging has proved very helpful in so many fields, such as cellular imaging, multimodal imaging, diagnosis of diseases, guided treatment, photodynamic and photo-thermal therapy, and drug delivery [381,383]. For the improvement of targeting strategies and specificity of PerLum nanoprobes, dual targeting imaging is a very attractive and effective tool due to the specific ligand-receptor interactions present on the exterior of a tumor cell. In 2016, Zhao et al. [362], synthesized ZnGa_2_O_4_:B^3+^, Cr^3+^ PerLum particles for dual-targeting cancer bio-imaging. For the improvement of specificity of PerLum nanomaterials for in-vitro and in-vivo bio-imaging with the help of bioconjugation of targeting legends present on the surface of such nanomaterials, which helps in dual targeting bio-imaging. As it has already been established that Cr^3+^ doping in ZnGa_2_O_4_ gives rise to near IR PerLum with a longer duration of afterglow [384], and this PerLum nanomaterial can be reactivated with the help of red light, using a co-doping of B^3+^ has been seen to improve the PerLum [362,385,386]. For the sake of using a dual targeting strategy with increased affinity and specificity, Zhao et al., used conjugation of hyaluronic acid and folic acid as targeting agents, as hyaluronic acid possesses specificity for cluster determinant 44 receptor and folic acid possesses specificity for folic acid receptor, which are present in cancerous cells. Thus, by using such a process, they could achieve dual targeting to attain the purpose of precise tumor targeting via the fabrication of PerLum nanomaterials [362].

**Fig. 14:**
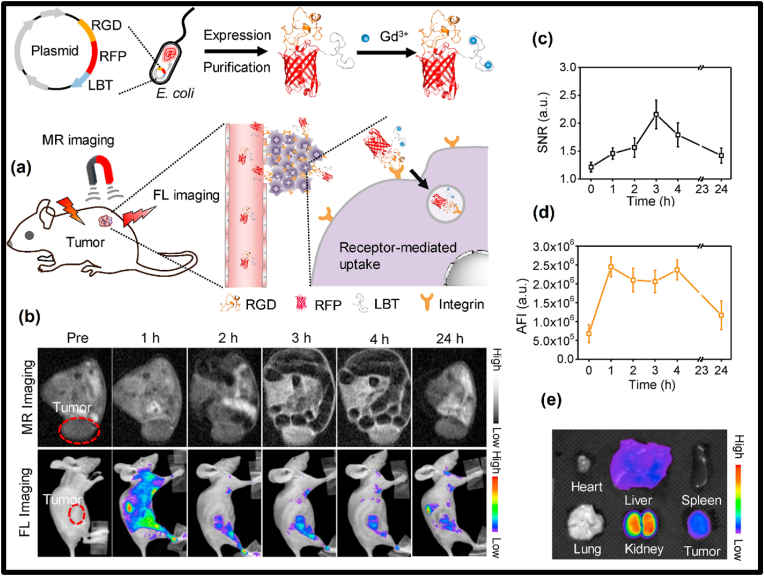
(a) Schematic diagram representing the general overview of biosynthesis of protein based molecular nanoprobe (RGD-RFP-LBT-Gd) with the application of tumour targeted fluorescence imaging (FL)/MRI. (b) MR/FL imaging of mice prior to injection and up to a duration of 24 h post injection with RGD-RFP-LBT-Gd. (c) Value of signal to noise ratio (SNR) of obtained MR images representing its variation with respect to time time. (d) Analysis of variation of intensity of FL signal (AFI) of the tumor at different intervals of time. (e) Representation and evaluation of biodistribution of RGD-RFP-LBT-Gd in different organs of a tumor bearing mice after a tail vain injection. Reproduced with permission from Ref. [387], Copyright 2020, Elsevier.

**Fig. 15:**
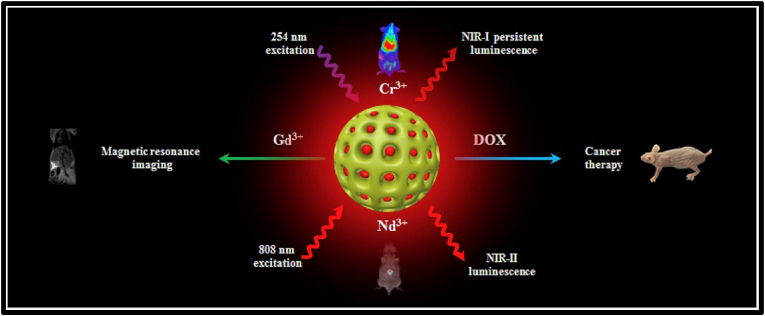
Schematic diagram representing the multimodal fluorescence imaging–MRI imaging strategy of PerLum based nanomaterials and use of such nanomaterials for cancer therapy. Reproduced with permission from Ref. [290], Copyright 2018, Elsevier.

**Fig. 16:**
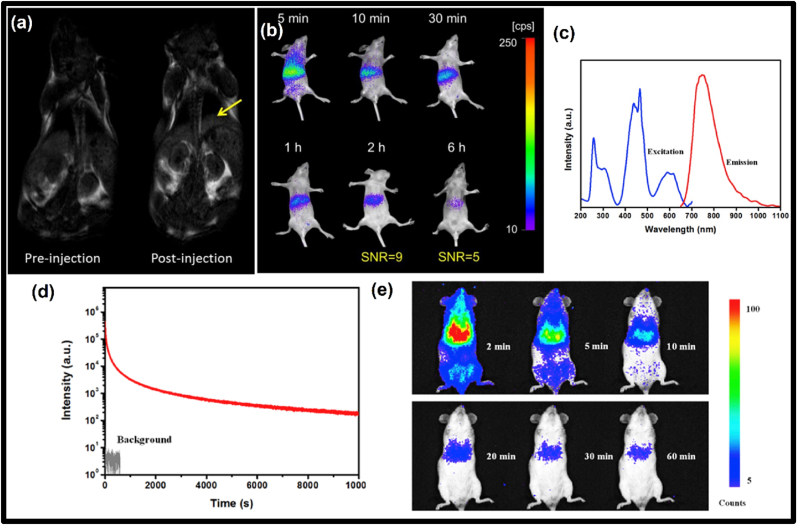
(a) Images displaying pre and post injection images of in-vivo T_1_ weighted MRI of mouse injected with Gd (III)-PerLum (yellow arrow points towards the liver) (b) in vivo fluorescence images of a normal mouse after intravenous injection of Gd (III)- PerLum nanomaterial. Reproduced with permission from Ref. [388]. copyright 2014, ACS Publications. (c) Figure showing excitation and emission spectra of mSiO_2_@Gd_3_Ga_5_O_12_ with peak emission at 745 nm and excitation peak at 254 nm (d) PerLum decay curve of mSiO_2_@Gd_3_Ga_5_O_12_ observed at peak emission of 745 nm 5 min post excitation via 254 nm UV photons. (e)in-vivo PerLum imaging of a normal mouse injected intravenously by mSiO_2_@Gd_3_Ga_5_O_12_ Reproduced with permission from Ref. [290], copyright 2018, Elsevier.

In year 2020, Zhao et al. [387], reported a protein-based nanoprobe for dual mode imaging based on fluorescence imaging and magnetic resonance imaging. They employed a biosynthetic approach by incorporating a targeting Arg-Gly-Asp (RDG) peptide with red fluorescent protein (REP) and a small peptide, which is a lanthanide-binding tag (LBT), with Gd^3+^, which possesses a strong affinity with this ion, as shown in Fig. 14 (a) [387]. Through the use of this probe, they observed highly sensitive imaging of tumors with enhanced specificity with the help of IR emission and improved tumor targeting. The strength of the signal was observed to attain a peak after 3 h of injection, after which the signal with high SNR lasted for about 1 h. They observed the intensity of MRI and fluorescence for 24 h after post-injection (Fig. 14 (b) [387]). The variation in SNR value and intensity of fluorescence signal with respect to time is shown in Fig. 14 (c, d) [387]. To observe the bio-distribution of the synthesized nanoprobes (RGD-REP-LBT-Gd), they collected fluorescence images of different organs and tumors after injecting them in the tail vein and observed a clear and strong signal in the liver and kidney, as shown in Fig. 14 (e) [387]. Apart from that, they also detected T_1_ weighted MRI both in-vivo and in-vitro, thus allowing imaging of tumors with high spatial resolution [387]. Medical diagnosis has been benefited by the use of multifunctional nanoparticles that use the advantages of both NIR fluorescence imaging and MRI with the help of PerLum nanoparticles, which have proved ideal candidates for optical imaging because of the absence of autofluorescence, high value of SNR, and deep tissue penetration. In 2018, Shi et al. [290], reported a novel PerLum nanomaterial, mSiO_2_@Gd_3_Ga_5_O_12_:Cr^3+^, Nd^3+^, for multimodal imaging and cancer therapy. They observed that these nanomaterials display the phenomenon of PerLum with afterglow for over 3 h in the wavelength range of 745 nm, thus making them a perfect candidate for increased, enhanced SNR and long-term in-vivo fluorescence imaging. They also observed that the high concentration of Gd^3+^ in the host Gd_3_Ga_5_O_12_ makes these nanoparticles suitable for T_1_ MRI, as shown in Fig. 15 [290]. Abdukayum et al.*,* [388] reported for the first time in 2014 PerLum-based multimodal nanoparticles, which were PerLum nanoparticles functionalized with gadolinium complexes and were used for the purpose of MRI and NIR-based fluorescence imaging. They observed that such PerLum-based nanoparticles presented improved longitudinal relativity than conventional Gd (III)-diethylenetriamine pentaacetic acid complexes, and because of the longer duration of afterglow, such nanoparticles offered a huge advantage in in-vivo fluorescence imaging and MRI. To analyze the efficiency of T_1_ weighted MRI, they injected mice with a Gd (III)-PerLum nanoprobe, and after comparing the pre- and post-injection images, they could observe that the liver of the mouse was clearly visible 15 min post-injection, which was not clearly visible as shown in Fig. 16 (a) [388]. This group also observed the in vivo NIR fluorescent imaging of mice post-intravenous injection of Gd (III) PerLum solution. Before injecting the mice with PerLum solution, it was excited for 10 min by the 254 nm UV lamp, and thus fluorescent images of nude mice with no in-situ excitation were obtained, as shown in Fig. 16 (b) [388]. Wang et al. [389], during the year 2017 also reported a multimodal nanoparticle based on functional composites of PerLum nanoparticles possessing NIR emission and Gd_2_O_3_ as a multimodal probe for displaying PerLum and thus being used for MRI as well. For the generation of hyaluronic acid (HA) functionalized multimodal probes, the technique used by this group for joining PerLum nanomaterials functionalized with amino groups and HA-Gd_2_O_3_ was the coupling strategy of N- (3-Dimethylaminopropyl)-N’-ethylcarbodiimide hydrochloride and N-hydroxysuccinimide. It was seen that this nano probe displays a strong NIR PerLum signal along with increased longitudinal relaxation of 7.38 mM^−1^s^−1^. They also observed that HA increases biocompatibility and provides the nano probe with the property of tumor targeting ability. Shi et al. [290], studied the NIR-I PerLum of mSiO_2_@ Gd_3_Ga_5_O_12_ and it was observed that such nanoparticles show a strong emission peak in the wavelength range of 745 nm when excited with UV photons of a wavelength of 254 nm, as shown in Fig. 16 (c) [290]. 5 min post-injection, the intensity of the signal at 745 nm excited at 254 nm could be detected with ease, as it was quite intense even after approximately 3 h of excitation, as shown in Fig. 16 (d) [290]. They also reported that the afterglow could be detected for up to 20 h of excitation, thus possessing good potential for in-vivo bio-imaging. They reported this strong emission to be related to the Cr^3+^ spin allowed transition from ^4^T_2_–^4^A_2_ [133]. The nanoparticles were injected into mice to analyze their ability for the application of in-vivo bio-imaging. After injecting mice with mSiO_2_@ Gd_3_Ga_5_O_12_ nanomaterial, the PerLum signal was received from the whole body for 5 min post-injection, as can be clearly observed from Fig. 16 (e) [290]. Zou et al. [390], reported in 2021 mesoporous silica based large pore nanoparticles synthesized via a template strategy. They reported these nanoparticles to be multifunctional hybrid mesoporous nanomaterials, which include the combination of Ga_2_O_3_:Cr^3+^, Nd^3+^-based PerLum nanoparticles with NIR emission, magnetic nanoparticles (Gd_2_O_3_), and ^68^Gd-based radionuclides. They reported these nanomaterials to have rechargeable NIR PerLum along with properties of strong radioactivity and enhanced longitudinal relativity. Because of such properties, such hybrid mesoporous nanoparticles could be used for long term NIR PerLum imaging, MRI, and position emission imaging (PET). Because of the use of nanoparticles with large pore sizes, it was found that these hybrid nanoparticles possess high drug loading capability [390]. Pei et al. [391], reported that nanomaterials doped with lanthanide ions possess an increased emission time in the wavelength range of second NIR windows ranging from 1000 to 1700 nm (NIR-II). These found that the use of core shell structured nanoparticles provides the advantage of tunability in NIR-II PerLum nanoparticles, which helps in improving the SNR ratio as compared to NIR-II fluorescence imaging along with accurate in-vivo multiplexed encoding and better resolution of abdominal vessels in mice. They also got four times more SNR value and three times more sharpness in the case of abdominal vessel images and deep tissue images of ureters (2–4 mm). With the help of such designed nanoparticles, they also observed multimodal NIR-II PerLum-MRI-PET imaging in the case of murine tumors and high contrast images of viscera [391]. In the pursuit of developing rechargeable PerLum properties for in vivo bioimaging applications Giordano et al. [392], synthesized and combined β-NaGd_0.8_Yb_0.17_Er_0·03_F_4_ nanoparticles displaying efficient upconversion with Zn_1.33_Ga_1.335_Sn_0·33_Cr_0·005_O_4_ nanoparticles having PerLum property via dry impregnation. They were successful in obtaining hybrid material exhibited persistent luminescence at 700 nm after excitation with a 980 nm laser.

One more imaging technique that has shown great potential in bio-imaging is called "multimodal imaging. This imaging technique is based on the integration of the advantages of various imaging modalities to generate images with increased sensitivity and improved resolution at the same time [393]. As we know, different imaging techniques show promising results in some areas while suffering from some limitations as well, so it is very appealing to integrate the unique properties of different PerLum nanomaterials with the unique abilities of various imaging techniques for the creation of high-performance PerLum-based imaging probes [388,394]. MRI has the ability to generate images with enhanced spatial resolution that are both anatomical and physiological in nature. Thus, if PerLum nanomaterials are incorporated with such an imaging technique, then a dual modal imaging nanoprobe can be designed [393].

#### 6.1.2 Per Lum-based image targeted therapy

Up until now, a lot of imaging techniques have been developed in order to monitor and visualize various life processes along with the diagnosis and progression of diseases, such as MRI, computed tomography (CT), optical imaging, etc. The field of bio-imaging has seen a lot of advancement, thus allowing the use of different diagnostic strategies with higher efficiency and very few side effects [361,395]. The use of PerLum for bio-imaging allows targeted imaging with the help of disease markers. Thus, imaging probes are the most vital components of modern-day targeted imaging and have helped in identifying the location of diseased tissue, thus providing improved diagnosis [302,[396], [397], [398]]. Research in bioimaging is thus focused on the development of improved and reliable probes in order to make this process easier and more efficient. In 2017, Zhang et al. [399], synthesized Zn_2_SnO_4_:Cr^3+^ co-doped with Eu^2+^ with a size of a few nanometers and used this PLNP for deep tissue imaging. They reported that this nanophosphor showed NIR luminescence with afterglow emission in the wavelength range of 800 nm, which was seen to easily penetrate up to 3 cm of pork tissue. They also observed a high SNR at the back of the mice, which could be maintained for a duration of 15 min. For accurate and precise diagnosis of tumor tissue, they conjugated these nano probes with folic acid for enhanced in-vitro and in-vivo targeting. To improve the quality of images, the use of multimodal imaging has proved beneficial as it provides multi-dimensional imaging data, thus enhancing the accuracy of diagnosis. In 2018, Liu et al. [400], synthesized ZnGa_2_O_4_:Cr^3+^ with a very high quantum yield of almost 20 %, strong NIR emission, and improved stability via the hydrothermal method. This PLNP displayed an afterglow of almost 120 h, and it could also be activated repeatedly at a wavelength of 657 nm. To enhance their stability and compatibility, these persistent nanophosphors were functionalized with poly (acrylic acid) and α, ω-dicarboxyl-ended poly (ethylene glycol), conjugated with c (RGDyK) peptide, and labelled with ^99m^Tc. They used this nanoprobe for phosphorescent in-vivo imaging and single photo-based CT. This method of imaging showed improvement by generating autofluorescence-free imaging with a high SNR. Wang et al. [286], synthesized Zn_1+x_Ga_2-2x_Ge_x_O_4_ doped with Cr (value of x ranging from 0 to 0.5) having the property of PerLum and composition dependent size, i.e., its size increases with an increase in the value of x. They observed that any variation in the value of x also helps to tune emission. These nanoparticles were observed to remove autofluorescence, and they showed long-term bioimaging. Such nanoparticles were easily activated in-vivo and, with the help of an aptamer-guided nanoprobe, accumulated with highly precise precision inside tumor tissue. By integrating the process of bio-imaging with guided therapy, the efficiency of cancer treatment can be greatly improved, and by combining the two on a single nanoplatform, high therapeutic efficiency can be achieved with minimal damage to healthy tissue. Simultaneous imaging and therapy have been accomplished via the use of complex nanostructures that act as imaging probes and delivery agents, but the main disadvantage with such nanostructures is that they need to be continuously excited throughout the whole procedure, which hampers their detection ability. Maldiney et al. [365], reported a novel PerLum based multifunctional agent composed of a Cr-doped zinc gallate core covered with a mesoporous silica shell and showed that such nanostructures can efficiently carry doxorubicin, which possesses specific cytotoxicity towards U87MGc cells. At the same time, the PerLum signal from doxorubicin-loaded mesoporous nanomaterials can be used for its improved in-vivo detection sensitivity, thus allowing real-time distribution with minimal to zero autofluorescence. This is because this guided process of treatment helps to monitor the release and distribution of drugs in the body for evaluation of therapeutic effects [365]. If a photosensitizer or a photo-thermal agent is mixed with imaging agents, then the irradiation of the tumor by the source of treatment can easily reveal its location, along with attaining the goal of photodynamic or photo-thermal therapy [367,401].

The use of PLNPs in this process is attributed to their extended afterglow time, which helps to eliminate autofluorescence for biomolecules present in the surrounding environment, and the tuning ability of such materials, which allows us to regulate the emission to a biologically transparent window for much deeper penetration, allowing deep tissue imaging and treatment [32,363]. The use of PerLum for bio-imaging and treatment of cancerous tissue can thus improve SNR and allow specific treatment. NIR-induced photo-thermal therapy is a non-invasive treatment technique that involves killing tumor tissue with the help of heat generated at the location of the tumor with the help of a photo-thermal agent and a NIR laser [402]. This technique has received a lot of attention for the treatment of cancer because of its non-invasive nature and deep tissue penetration [403,404]. Targeted photo-thermal therapy assisted by bio-imaging is helpful compared to conventional photo-thermal therapy as it does not require a broad range of the NIR spectrum and it provides targeted images of tumor tissues, thus providing a precise target for NIR irradiation [368,401]. Chen et al. [368], reported the design of a multifunctional PLNP, CuS based nano probe for guided in-vivo photo-thermal therapy and for connecting PerLum nanophosphor to copper sulphide in order to build an activatable nano probe and they have used a matrix metalloproteinase (MMPs)-specific peptide substrate (H_2_N–GPLGVRGC–SH). They observed that this nano probe offers a very low background for in-vivo luminescence because of its zero autofluorescence and activatable property and, is very effective for photothermal therapy because of the presence of copper sulphide nanoparticles.

**Fig. 17:**
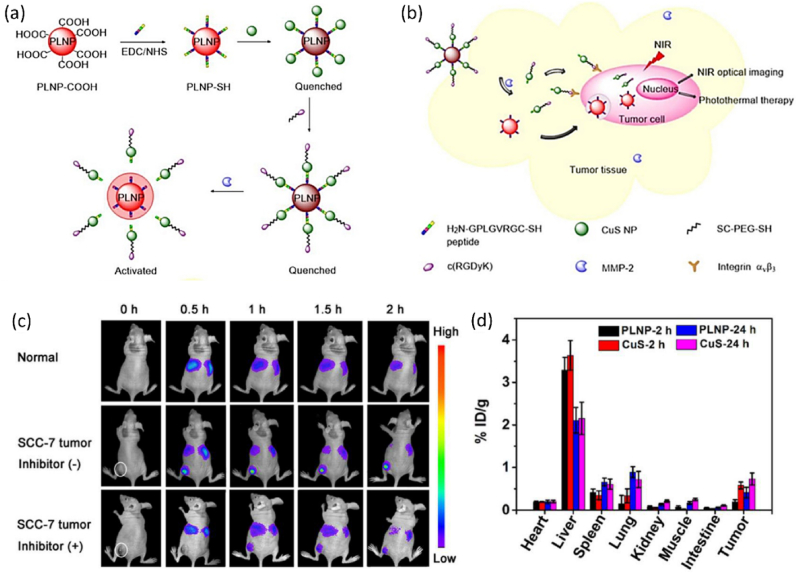
(a) Pictorial representation of design, synthesis and activation of PLNP-CuS-RGD nanoprobe (b) schematic representation for use of PLNP-CuS-RGD nanoprobe for in-vivo image guided photothermal therapy. (c) in-vivo bioimaging of mice with SSC-7 tumor after injecting them with PLNP-CuS-RGD nanoprobe (intravenous) showing images obtained in presence and absence of inhibitor (d) representation of biodistribution of PLNP and CuS by SSC-7 tumor present in different organs of the tumour bearing mice at after 2 h and 24-h time interval after the intravenous injection. Reproduced with permission from Ref. [368], copyright 2016, ACS Publications.

Fig. 17 (a) [368] represents the design and scheme for the preparation of a multifunctional and reusable CuS-based nano probe in which the activation property is attributed to PLNP and CuS. In this nano probe, CuS acts as a photo-thermal agent and NIR quencher, thus generating improved photo-thermal efficiency [405]. To activate the nanoprobe luminescence in the presence of MMP, this research group took advantage of the H_2_N–GPLGVRGC–SH substrate for linking luminescent nanoparticles with copper sulphide nanomaterials. The used peptide functionalized into PerLum nanomaterial was synthesized by the process of condensation of NH_2_ and carboxylic acid groups in the peptide substrate and PerLum nanomaterial, respectively. The formation of activatable nano probe is thus assisted by the terminal SH group obtained from PLNP functionalized with peptide substrate and used to conjugate copper sulphide nanoparticles. The binding of PEG and c(RGDyK) groups was also done for improving targeting ability and biocompatibility. Fig. 17 (b) [368] shows that in the synthesis of activatable nano probe for in-vivo bio-imaging and photo-thermal therapy, first SC−PEG−SH and NH_2_ groups were bound via a covalent bond, and after that, they were joined with copper sulphide. The ability to perform in-vivo bio-imaging with the help of the PLNP-CuS-RGD nano probe was then analyzed by injecting (intravenous) this photoluminescent nano probe along with and without the presence of an inhibitor into a mouse with MMP-2 enzyme positive (SCC-7) tumor. In-vivo images were then captured from these mouse subjects, which were obtained by illumination of this nanoprobe with 254 nm UV-radiation for 10 min before injection and illumination for 2 min by 650 nm LED light prior to every acquisition, as represented in Fig. 17 (c) [368]. For the analysis of the distribution of this nano probe inside the test subject, concentrations of zinc and copper from PLNP and copper sulphide were analyzed in different organs, and as shown in Fig. 17 (d) [368], it can be seen that retention of the used nano probe increases with time in the SCC-7 tumor. Because of the large accumulation of PLNP−CuS−RGD inside the liver, which is a major reticuloendothelial organ, a detectable signal was produced from the liver for half an hour after injection because of incomplete quenched luminescence, but after a time of 2 h, the signal strength had decreased at a quick rate, thus producing a low probe background signal. Another approach to treating and curing cancer is called photodynamic therapy, which involves the use of a photo reactive molecule called a photosensitizer [[406], [407], [408], [409], [410]]. Upon exposure to photons of a suitable wavelength, this photosensitizer generates singlet oxygen, which possesses the ability to kill cancer cells directly, destroy the vasculature of the tumor, and thus generate an immune system against further growth of the tumor [411]. Because of the improved efficiency and lower toxicity of this technique, it has received a lot of attention in recent years [367,412]. One major limitation of this technique is the use of high-frequency excitation wavelengths, which may cause damage to healthy cells, photo allergic damage, and, in some cases, even genetic mutations. Thus, it is advisable to use targeted photodynamic therapy with the help of PerLum bio-imaging. PerLum based bio-imaging-guided photodynamic therapy can give rise to accurate targeting of cancerous tissue with the help of an imaging nanoplatform and thus irradiation of that precise location [413].

**Fig. 18:**
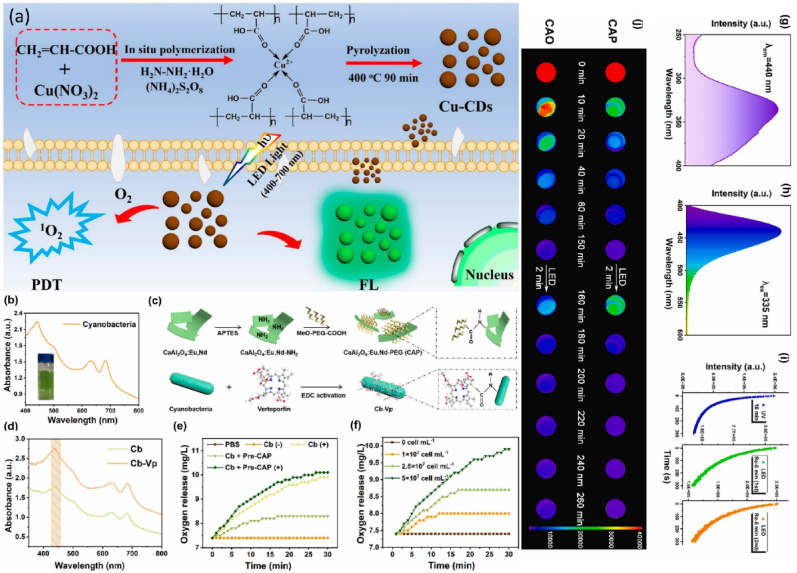
(a) Schematic diagram representing the process of synthesis of copper doped carbon dots, their property of fluorescent imaging and their application as bio-imaging guided photodynamic therapy Reproduced with permission from Ref. [414], copyright 2019, Elsevier. (b) UV–Vis absorption spectrum of cyanobacteria (Cb) representing absorption peaks of 440 nm, 630 nm and 681 nm (c) schematic diagram representing the fabrication of CaAl_2_O_4_: Eu, Nd and design of cyanobacteria-verteporfin (Cb-Vb) (d) Absorption spectrum of cyanobacteria and cyanobacteria-verteporfin combination for calculation of verteporfin loading efficiency. (e) Variation in dissolved oxygen level of the prepared solution regarding different treatments where (−) means absence of irradiation and (+) means presence of irradiation. (f) Variation in the level of dissolved oxygen of Cb with variation in the density of cells under irradiation because of white LED. (g) Excitation spectrum of CaAl_2_O_4_ (h) Emission spectrum of CaAl_2_O_4_ (i) decay curves of CaAl_2_O_4_ after repeated excitation with white LED lamp (j) afterglow luminescence images of CaAl_2_O_4_: Eu, Nd (CAO) and pegylated CaAl_2_O_4_: Eu, Nd (CAP) after 10 min irradiation with photons of wavelength of 365 nm UV lamp and re-activation by white LED for a duration of 2 min [415], Copyright 2022, Elsevier.

This technique has the dual advantage of improved efficiency and minor collateral damage. In 2017, Chen et al. [413], used X-ray as an activation wavelength for photodynamic therapy. They synthesized a novel LiGa_5_O_8_:Cr based PerLum nano-scintillator with NIR luminescence in the X-ray wavelength range. Because X-ray is transparent to tissue, it could be used for deep tissue optical imaging and thus for guided irradiation. Chen et al. [413], synthesized the nano-sanitizers by loading 2,3-naphthalocyanine and LiGa_5_O_8_ doped with Cr^3+^ onto mesoporous silica nanoparticles. Among these two, naphthalocyanine could be used for photodynamic therapy as it can generate singlet oxygen used to kill tumor cells, and LiGa_5_O_8_ doped with chromium acts as an imaging agent with the properties of PerLum. Due to the in-vitro excitation of LiGa_5_O_8_, it becomes possible to generate images with a SNR and autofluorescence-free in-vivo images. Because of this property, it is possible to specifically target cancer cells, thus improving the therapeutic effect of singlet oxygen produced by naphthalocyanine and minimizing the collateral damage caused by X-ray irradiation. Wang et al. [414], used copper doped carbon dots for optical bio-imaging and subsequently targeted photodynamic therapy. They synthesized copper-doped carbon dots with a quantum yield of 24.4 % from polyacrylic acid copper complexes via the process of coordination between copper ions and the carboxyl group. Because of their strong luminescence and low cytotoxicity, carbon dots could be easily used for bio-imaging in human cervical cancer and human neuroblastoma cells. One more advantage of using such luminescent materials is that their singlet oxygen yield is approximately 36 %. Because of this, copper-doped quantum dots can prove promising candidates for photodynamic therapy. As shown in Fig. 18 (a) [414], copper-doped quantum dots were synthesized via in-situ polymerization of acrylic acid and copper nitrate as precursors, ammonium persulfate, and hydrazine as initiators and reducing agents, respectively. After pyrolyzation at 900 °C for 90 min, the product formed were, earlier Cu-doped carbon dots. They observed that these quantum dots could be used for bio-imaging-guided photodynamic therapy because of their properties of fluorescent imaging and the generation of singlet oxygen. Chang et al. [415], reported a novel design of CaAl_2_O_4_:Eu co-doped with Nd and blue PerLum in 2022 for reducing cytotoxicity due to long-term irradiation as well as decreasing hypoxia. They found that this nanophosphor possesses the characteristics of an optical battery triggers verteporfin and cyanobacterial cells together. Because of this successive generation of singlet oxygen, there is no kind of long-term excitation, thus resulting in improved and enhanced photodynamic therapy. Fig. 18 (b) [415] represents the absorption spectrum of cyanobacteria, and, as seen in the same Figure, the strong peaks of 440, 630, and 681 nm are there because of the presence of chlorophyll [416]. The combination and suitable design of cyanobacteria-verteporfin and CaAl_2_O_4_:Eu co-doped with Nd was performed by them for the application of minimizing tumor tissue and performing photodynamic therapy with high specificity, as clearly shown in Fig. 18 (c) [415]. Absorption spectra of verteporfin for different concentrations ranging from 0 to 10 μg m/L and for the calculation of the loading capacity of verteporfin standard concentration curve was calculated at 2.99 wt %. They analyzed the absorbance difference of cyanobacteria and a combination of verteporfin and cyanobacteria at a wavelength peak of 431 nm, thus showing the successful loading of the photosensitizer as shown in Fig. 18 (d) [415]. Without the addition of cyanobacteria, no fluctuation effect was observed in phosphate buffer solution (PBS) with a pH of 7.4, thus showing the equilibrium of dissolved oxygen as illustrated in Fig. 18 (e) [415]. The difference between levels of dissolved oxygen in irradiated and non-irradiated cyanobacteria with the help of a white LED, represented as Cb (+) and Cb (−), respectively, represents the photosynthetic oxygen induced by a white LED lamp. Fig. 18 (f) [415] illustrates the results of the density correlation oxygenation experiment, confirming the fact that the concentration of dissolved oxygen depends on the concentration of cyanobacteria, thus showing its importance for tumor treatment. For guided irradiation, it is imperative to make use of PerLum from CaAl_2_O_4_:Eu co-doped with Nd, as it helps to specifically target the tumor cells. Chang et al., observed the excitation spectrum at 440 nm wavelength, as shown in Fig. 18 (g) [415], which shows a peak excitation band at 335 nm in the broad excitation spectrum ranging from 300 to 400 nm. Because of the peak excitation at 335 nm, the emission spectrum was observed at the same wavelength for the used PerLum nanomaterial, as shown in Fig. 18 (h) [415]. The broad emission spectrum in the wavelength range of blue light (400–500 nm) of this PerLum nanomaterial is because of the 5d–4f optical transition. Optical battery characteristics of this synthesized nanomaterial can be verified via its reactivation by a white LED lamp, and thus blue luminescence was captured for 5 min after re-irradiation (Fig. 18 (i) [415]). For the improvement of biocompatibility, surface functionalization of CaAl_2_O_4_ with polyethylene glycol (CAG) was performed, and it was observed that this modification has no significant effect on the luminous intensity and afterglow time of CaAl_2_O_4_, as understood from Fig. 18 (j) [415]. From the above discussion, afterglow generated due to PerLum nanomaterial can improve the efficiency of photodynamic therapy, improve autofluorescence, enhance SNR, and allow long term in-vivo bio-imaging. The emission from such a nanomaterial can be regulated in such a way that it lies in the biologically transparent window, allowing deep tissue penetration. Because of such properties, these nanoparticles have been widely used for applications in disease diagnosis, image-guided photodynamic therapy, image guided photothermal therapy, and drug delivery [44].

#### 6.1.3 Per Lum based biosensing

PerLum based biosensors are certain materials that change their luminescent properties, such as intensity, decay time, or emission spectrum, in response to an analyte of interest, thus providing data about their presence and their quantitative changes with time [241,417]. They generate luminescence of a certain intensity when they come in contact with one of the most effective ways to remove autofluorescence interference is time-resolved luminescent biosensing [397,[418], [419], [420], [421]]. However, this method frequently requires the use of complex and expensive tools. PerLum nanomaterials possess an advantage here because of their extremely long afterglow; they can prevent autofluorescence in a similar but more straightforward manner. In this part of the review, we will discuss some important facts and groundbreaking work in this field. The detection of fluorescence, which has the characteristics of high sensitivity and convenience of use, is becoming more and more popular in basic research and clinical practice. Owing to imperfections from autofluorescence and scattered light in biological matrixes under continual external stimulation, typical fluorescent probes have a low SNR ratio. Extended afterglow allows the excitation of the nanomaterial outside the host body, thus removing the chances of autofluorescence and helps in resolving the problem of high background signal in the case of fluorescent biosensing [353,355].

**Fig. 19:**
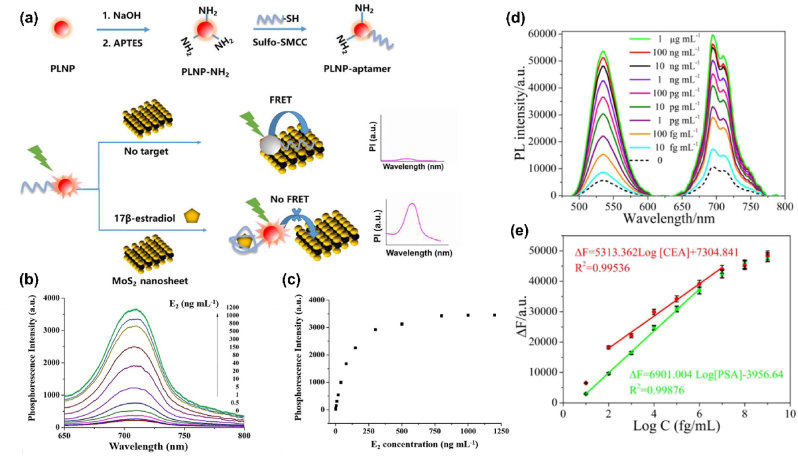
(a) Mechanism of aptasensor luminescence because of 17β-estradiol Foster resonance energy transfer (FRET) between MoS_2_ nanostructures and aptamer-functionalized PLNPs. (b) The effect of 17β-estradiol concentration on PLNP functionalized-aptamer/MoS_2_ luminescence. (c) Luminescence spectra of estradiol standard solution at different concentrations. Reproduced with permission from Ref. [422], copyright 2022, Elsevier. (d) Luminescence spectrum of the apta-sensors for detecting varied prostate-specific antigen (PSA) and carcinoembryonic antigens (CEA) concentrations. (e) Linear correlations between fluorescence intensity (F–F_0_) and PSA (green) and CEA (red) concentrations, ΔF = F–F_0_, F denotes intensity in the availability of targets, F_0_ denotes intensity in the absence of targets. Reproduced with permission from Ref. [422], copyright 2020, ACS Publications.

Recently, Zhang et al. [422], reported NIR PerLum nanoprobes for background signal-free 17β – estradiol in milk samples. For this expected biosensing, they synthesized a NIR PerLum apta-sensor for its detection with no autofluorescence. They reported that this biosensor was based on Foster Resonance Energy Transfer (FRET) between MoS_2_ nanosheets and PLNPs. The detection of the 17β–estradiol was based on the analysis of afterglow from the synthesized PerLum nanoprobes, by which as low as 0.29 ng mL^−1^ concentration was detected in a 0.5 mL sample. In 2017, Wang et al. [423], reported 1–D nanorods with the property of PerLum for the application of autofluorescence free biosensing. They reported the direct synthesis of Zn_2_GeO_4_:Mn (ZGO:Mn) via the hydrothermal synthesis method. They observed that the length and PerLum of these nanorods could be easily varied by a simple variation of pH during synthesis, and hydrothermal reactions also allowed rapid growth and intense PerLum just after 30 min of hydrothermal treatment. They further synthesized aptamer-guided ZnO:Mn and used it for the analysis of serum lysozyme. The amounts of lysozyme in serum samples taken from individuals with colorectal cancer, stomach cancer, and lung cancer were determined. They observed that background interference in the serum was completely removed because of the longer afterglow of these bio-probes. The bio-probe lysozyme measurement findings were consistent with the results obtained using a clinical approach, showing that they have a lot of potential in clinical sample analysis. Using a solvothermal technique of synthesis, Hu et al. [424], *in the* year 2019 reported the synthesis of Zn_3_Ga_2_GeO_8_:Cr (ZGGO:Cr) PLNPs with the tunability property of size and extended afterglow. They observed that the dimensions of the developed PLNPs rapidly declined as the pH level is increases in the solvothermal synthesis method. As the pH value rises, the duration of the afterglow increases and the intensity of PerLum decreases. They reported the use of such nanoparticles for the measurement of lifetime of different biological analytes. Glutathione (GSH) sensing was accomplished by utilizing ZGGO:Cr@MnO_2_ PLNPs with a core-shell structure. The content of GSH can be measured by observing the PLNPs' prolonged afterglow after the termination of irradiation. Determination of lifetime via ZGGO: Cr@MnO_2_ nano probe possesses the advantage of high sensitivity and improved selectivity for GSH. They observed that this synthesized nano probe possesses an extended afterglow and size, that can be varied regarding the pH of the surrounding environment, thus showing that it can be used for autofluorescence-free biosensing. Because of the non-requirement of *in situ* excitation and the increased value of SNR, PerLum-based nanoprobes have been receiving more and more attention in recent times for the application of biosensing. The Property of afterglow in PerLum materials can also be of particular interest for the development of in vitro biosensors. Liu et al. [425], reported that hydrogen peroxide (H_2_O_2_) increases the signal intensity of ZnGa_2_O_4_:Cr^3+^ nanoparticles. They suggested that this signal amplification could be used to detect and quantify H_2_O_2_ in various media using non-functionalized ZnGa_2_O_4_ nanoparticles. Zhang et al. [422], observed that PLNP-aptamer is adsorbed with the help of MoS_2_ nanostructures in immediate contact if 17β-estradiol, is not present, as illustrated in Fig. 19 (a) [422], due to which luminescence from excited PLNP is suppressed because of MoS_2_ nanostructures. Because of the strong binding tendency between 17β-estradiol and PerLum aptamers, this aptamer nanocomposite bonds to substrates when 17β-estradiol is present, thus releasing PLNP from nanostructures and giving rise to luminescence. A change in luminescent intensity thus shows 17β-estradiol, whereas a variation in intensity shows a quantitative change in estradiol. To investigate the responsivity of the luminous apta-sensor, they used varied quantities of 17β-estradiol (0.5–1200 ng mL^−1^) in the biosensor under ideal circumstances, and evaluated the intensity of afterglow luminescence at the peak emission wavelength. They observed that the intensity of afterglow was rising steadily because of the increase in 17β-estradiol levels, as illustrated in Fig. 19 (b) [422]. Zhang et al., performed piecewise regression analysis because of segmentation attributes in the scatter graph of elongated concentration levels (Fig. 19 (c) [422]). In the year 2020, Shi et al. [426], attempted to synthesize tunable PerLum nanoprobes with expected de-excitation patterns for their use in biosensing with time-resolved fluoro-immuno assays. They synthesized nanorods based on the host material Zn_2_GeO_4_ and doped them with Mn^2+^, Mo^6+^, Cr^3+^, and Sr^2+^ with the property of PerLum for attaining the special de-excitation property. The biosensing probes used by them for detection of carcinoembryonic antigens and prostate-specific antigens were ZnGa_2_O_4_ doped with Mn and Cr with green emission, lower decay rates, and non-interfered colors. By using such nano probes, they could detect concentrations of carcinoembryonic antigens and prostate specific antigens as low as 72 fg mL^−1^ and 8.9 fg mL^−1^, respectively, and thus they could detect carcinoembryonic antigen concentrations in human serum matrix. They argued that this research opened up a new way for the fabrication of multifunctional PerLum nanoparticles with specific decay characteristics, which were previously only employed in the early detection of cancer and time-resolved fluoro-immuno assay. They observed that fluorescence intensity grew gradually as the number of targets increased, as seen in Fig. 19 (d) [426]. ΔF (F–F_0_) vs. logarithm of concentration of PSA was then plotted, with concentrations ranging from 10 fg ML^−1^ to 1 ng mL^−1^. With a linear correlation value of 0.99876, the detection limit was estimated to be as minimal as 8.9 fg mL^−1^. A direct correlation among F–F_0_ and CEA concentration was framed for a concentration range of 100 fg mL^−1^ to 10 ng mL^−1^, with a linear correlation value of 0.99536, and a detection limit of 72 fg mL^−1^ (Fig. 19 (e) [426]).

### 6.2 Security applications of persistent luminescent nanomaterials

During the current decade and for some time now, crimes involving thefts, terrorism, murder, counterfeiting, etc., have increased at a quick rate. Thus, it has become imperative to develop futuristic security applications. In this review, we will focus on applications of PerLum nanomaterials for anti-counterfeiting and latent fingerprinting. Copyright infringement or counterfeiting of sensitive documents, banknotes, and premium brands is a tricky issue with major financial, security, and medical impacts for government agencies, corporations, and individual consumers around the world. It is considered a multi-billion-dollar illegal sector that produces millions of counterfeited products every year. It has been observed that this crime is getting more and more high-tech with every passing day, and thus the development of highly secure and high-tech systems for anti-counterfeiting is the need of the hour. Because of the wide bandwidth, low cost, agility, and stability, luminescent nanomaterials have been used at a very large scale in the anti-counterfeiting industry [427]. Most contemporary luminescent nanomaterial-based anticounterfeiting systems rely on down-conversion or up-conversion, where continuous excitation is needed; e.g., Chinese and Euro currency notes both make use of lanthanide fluorescence to combat counterfeiting [[428], [429], [430]]. But as already discussed, continuous activation leads to the generation of background signals and interference [431] thus, the use of PerLum for anticounterfeiting technology that has the potential to completely remove background signals is critical and strongly sought after. Latent fingerprints (LFPs) could act as "identification cards" and "data archives" because of their unique arrangement and rich chemical makeup, making them perfect for forensic analysis, security systems, and even diagnostics. Analysis of latent fingermarks has received a lot of attention from researchers, and thus a lot of different materials have been synthesized for enhancing fingerprint detection. One of the major problems with LPF is the presence of a background signal in the final output because of which LPF images appear blurred and distorted, thus reducing imaging resolution. Hence, efforts need to be made to reduce the background signal, which brings us to the phenomenon of PerLum. Particularly elevated areas of the epidermal layer of skin on a person's fingertips and palms have been observed to generate skin ridge formations which post analysis can be used as key identification markers [432,433]. The most important advantage that makes them imperative and highly useful for forensic examination is that they possess the advantage of being genetically distinct and permanent [432,434], allowing us to use them for a variety of security applications such as data encryption, criminal investigation, and authoritative enforcement [435].

#### 6.2.1 PLNPs for anti-counterfeiting

As already discussed, PerLum does not need continuous irradiation for emitting photons, so its use for anti-counterfeiting can lead to zero autofluorescence and thus efficient results and better anti-counterfeiting. Liu et al. [436], synthesized metal-organic frameworks (MOFs) with stable structure, enhanced lifetime, and longer afterglow in 2018 by encapsulating various dyes into luminescent MOFs with green-colored emission. They observed that MOF-to-dye luminescent transfer of energy generates multicolored emission with enhanced wavelength and a longer afterglow possessing an emission wavelength ranging from green to red. They argued that such remarkable phosphorescent MOFs with increased lifetime could be used as ink pads for enhanced anticounterfeiting because of their exceptional optical features. Because of these properties of MOFs, this research not only provides a gateway for the creation of novel luminescent materials with increased lifetime, multicolored emission, and longer afterglow, but could also be used for the development of improved luminescent anticounterfeiting materials. In the year 2019, Ji et al. [437], reported a PerLum phosphor Sr_2_Ga_2_GeO_7_ doped with Pr^3+^ with multicolor emission. They reported that the color of emission of this luminescent material could be adjusted from dark red to blue. This color variation was reported to be caused in Sr_2_Ga_2_GeO_7_:Pr^3+^ phosphor due to be presence of cross-relaxation of Pr^3+^ in the host lattice, which possesses low phonon-assisted energy. This research group was also able to adjust the afterglow time of this phosphor, and after observing some PerLum photographs it was observed to reveal dynamic multicolor data based on unique afterglow features of this phosphor. Such properties thus established that the PerLum images can represent dynamic multicolor anticounterfeiting thus making them fit for such applications. Single-mode luminescence and up-conversion phosphorescent compounds, which are primarily employed in single-level, monochromatic type of emission have also been very widely used for anti-counterfeiting applications.

Gao et al. [438], used multi-mode Zn_3_Ga_2_GeO_8_:Cr^3+^ co-doped with Yb^3+^, Er^3+,^ and Zn_1·6_Li_0.4_GeO_4_ doped with Mn^2+^ PerLum nanophosphors as the fluorescent inks to attain quintuple-mode, multi-color dynamic anti-counterfeiting. The Zn_3_Ga_2_GeO_8_:Cr^3+^ co-doped with Yb^3+^, Er^3+^ was observed to possess quintuple phosphorescent modes, PerLum, photo-stimulated phosphorescence, photo-stimulated PerLum, and UC phosphorescence with multi-color luminescence, whereas the Zn_1·6_Li_0.4_GeO_4_: Mn^2+^ phosphor possesses quadruple luminescence modes, PerLum, photo-stimulated phosphorescence, and photo-stimulated PerLum, having a long-lasting afterglow with green emission. Gao et al. [438], employed such materials to obtain dynamical pattern of emission colors and intensity on a dragon on lotus pattern and argued that this study can help in designing highly advanced strategies for optical anti-counterfeiting techniques. PerLum is a very attractive phenomenon where luminescent ink, records a change in time after the excitation source is switched off. During the year 2021, Katumo et al. [439], synthesized Gd_2_O_2_S:Eu^3+^/Ti^4+^ phosphor material on which the duration of afterglow could be modulated from 1.17 ± 0.02 s to 5.95 ± 0.07 s depending on the Ti^4+^ doping concentration. The luminescence quantum yield was observed to maintain at 46 % ± 3 % while tuning the PerLum lifetime. Such luminescent materials could be activated by employing affordable and relatively harmless LEDs with an emission wavelength of 375 nm due to the presence sufficiently large charge-transfer spectrum. Dynamic variations in the luminescent ink after switching off the excitation source were noticed by generating sequences with the help of phosphors that have different afterglow times. These changes were found to be visible to the naked eye in images constructed with the help of such phosphor materials, possessing large variations in afterglow duration. The variations in color patterns created by luminescent materials with almost equivalent persistent afterglow (0.20 s difference in luminescence afterglow time) were found to be challenging to see with the naked eye but could be clearly identified by examining a 30-frames-per-second video captured by a smartphone. As a result, such intense phosphors with configurable afterglow times could be used to both overt and covert anticounterfeiting identifiers. Because of the remarkable characteristics of optical accessibility and excellent stealth properties, luminescent substances play a critical role in anticounterfeiting technologies. while conventional luminous substances frequently display monochromatic emission and hence tend to be more frequently counterfeited. Cai et al. [440], reported a series of Zn_1+x_Ga_2−2x_Sn_x_O_4_ (ZGSO) nanoparticles with persistent luminescence in the near-infrared (NIR) I and II windows. When doped with Cr^3+^ and Ni^2+^, these nanoparticles exhibited intense photoluminescence under UV radiation, covering the red, NIR-I, NIR-II, and even NIR-III regions. They also showed persistent luminescence at 700 nm (NIR-I) and 1300 nm (NIR-II) after excitation removal. These properties make ZGSO nanoparticles a promising material for multi-level anti-counterfeiting applications. Pei et al. [441], in the year 2021 synthesized a multicolor emitting PerLum phosphor, NaCa_2_GeO_4_F, and doped it with different concentrations of Tb^3+^, where the color of PerLum emission could be adjusted from blue emission to cyan emission and bright green by changing the amount of Tb^3+^, and with 1, 2, and 3 mol percent of Tb^3+^, the afterglow could last for 5.62 h, 8.52 h, and 7.14 h, respectively. They reported that this polychromatic PerLum is primarily related to the trapping and cross-relaxation of Tb^3+^ in NaCa_2_GeO_4_F. Anticounterfeiting systems have been constructed based on the distinctive properties of PerLum, and the findings show that the polychromic nature of the emission can be easily identified with a UV lamp, making it very difficult to counterfeit such materials. Because of their improved resolution and ease of detection by directly viewing the color spectrum, fluorescent anti-counterfeiting substances have recently received a lot of attention. Single UV activation mode anti-counterfeiting techniques suffer from vulnerability, as they can be easily counterfeited because of the presence of single mode emission.

**Fig. 20:**
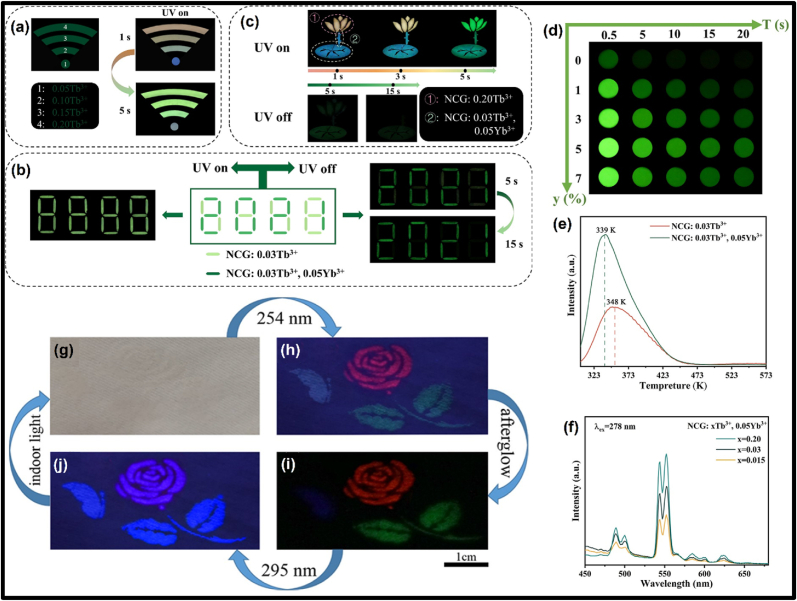
(a) Photographs of Wi-Fi pattern with diverse excitation time and the Wi-Fi pattern made of Na_2_CaGe_2_O_6_ with different concentration of Tb^3+^(b) afterglow images of a pattern made of Na_2_CaGe_2_O_6_ 0.03 Tb^3+^ and Na_2_CaGe_2_O_6_:0.03 Tb^3+^,0.05 Yb^3+^ (with intense and longer afterglow) excited with 254 nm wavelength for time of 5 min (c) shows anti-counterfeiting with the help of bi-temporal model using multi color afterglow and intense green Per Lum. (d) photograph representing afterglow of Na_2_CaGe_2_O_6_: 0.03 Tb, yYb^3+^ (y = 0,0.01,0.03,0.05,0.07) excited with the help of 254 nm excitation source for a time interval of 5 min. (e) graph represents Thermoluminescence glow curves of Na_2_CaGe_2_O_6_: 0.03 Tb^3+^ and Na_2_CaGe_2_O_6_: 0.03 Tb^3+^, 0.05 Yb^3+^ (f) graph representing photoluminescence spectra of Na_2_CaGe_2_O_6_: x Tb^3+^, 0.05 Yb^3+^(x = 0.015,0.03,0.20) recorded after being excited by photons of wavelength of 278 nm. Reproduced with permission from Ref. [443], copyright 2022, Elsevier. (g–i) Dynamic anti-counterfeiting images with help of polychromatic material composed of three parts butterfly, flower and leaf pattern with different dopant – co-dopant combination under where (g) indoor light (h) 254 nm excitation (i) after removal of excitation source (after-glow) and (j) 295 nm excitation source. Reproduced with permission from Ref. [444], copyright 2019, ACS Publications.

Zhu et al. [442], reported in the year 2022 a phosphor exhibiting PerLum with improved afterglow, photo-stimulated, and up-conversion properties of emission. They synthesized Ba_2_Zr_2_Si_3_O_12_ phosphor with doping of Eu^2+^ and Er^3+^ and, with its help, they were able to effectively create four modes of anti-counterfeiting. They observed that this phosphor could emit a strong blue-green illumination with a long afterglow attributed to the typical crossover of Eu^2+^ ions. Because of the introduction of Er^3+^ ions, the amplification of photo-stimulated phosphorescence attributed to increase in trap density is detected, which also gives rise to the phenomenon of UC. Under the illumination of a laser source employing an excitation wavelength of 980 nm, the luminescence was observed to swiftly transform from blueish-green to solid-green luminescence with the lag in the activation period. Pei et al. [441], observed that the quick shift from down-conversion to UC is the underlying phenomenon that makes this phosphor useful for high-tech multimodal anti-counterfeiting. Jin et al. [443], in the year 2022 reported a phosphor Na_2_CaGe_2_O_6_:Tb^3+^ co-doped with Yb^3+^ through which they could take advantage of its bright luminescence and time-varying emission to be used for dynamic and bi-temporal phosphorescent emission performance for more secure anti-counterfeiting thus allowing better and more dynamic anti-counterfeiting, PerLum offers a significant breakthrough to strengthen the field of information security. The cross-relaxation mechanism amongst Tb^3+^ ions lead to continuous alteration of the emission color of Na_2_CaGe_2_O_6_:Tb^3+^ from red-colored photons to green within a time interval of seconds with the help of UV excitation. The novel trapping technique through co-doping of Yb^3+^ was observed to improve the characteristics of PerLum in Tb^3+^-doped Na_2_CaGe_2_O_6_. Such findings provide an efficient technique for developing high-tech dynamical anticounterfeiting and data encryption systems, as shown in Fig. 20(a–c) [443]. In the Fig. 20 (a) [443], it can be clearly seen that by varying the concentration of Tb^3+^ ions, the dynamic variation in the emission color can be observed which can thus easily have employed for dynamic anti-counterfeiting. The WIFI pattern exhibiting variation in emission intensity and color was fabricated via the use of four different concentrations of Tb^3+^, represented as 1, 2, 3, and 4 by the use of Na_2_CaGe_2_O_6_:xTb^3+^ phosphor with x = 0.05, 0.10, 0.15, and 0.20, respectively. Jin et al. [443], reported that use of co-doping the Yb^3+^ employed for improving the afterglow of Na_2_CaGe_2_O_6_:Tb^3+^ along with, for improved dynamic anti-counterfeiting, which is because of a difference in afterglow duration between Na_2_CaGe_2_O_6_:Tb^3+^ and Na_2_CaGe_2_O_6_:Tb^3+^, Yb^3+^ (Fig. 20 (b) [443]). Here, the "2021' design was created by Na_2_CaGe_2_O_6_: 0.03 Tb^3+^, 0.05 Yb^3+^, and the rest by the use of 0.03 Tb^3+^ in the same phosphor. This design generates green emission with '8888' visible in it when exposed to UV light at 254 nm. Because of the variation in the afterglow duration between only 0.03 Tb^3+^ and 0.05 Yb^3+^, and only 0.03 Tb^3+^ doping in the used phosphor was observed to be responsible for generating the design of '2021' that forms steadily after the excitation source is switched off. It can also be observed that the use of PerLum with dynamic and multicolor emission can be used for optical anti-counterfeiting. As shown in Fig. 20c [443]; the emission color from the images of lotus flowers with Na_2_CaGe_2_O_6_: 0.20 Tb^3+^ and lotus leaves with Na_2_CaGe_2_O_6_: 0.03 Tb^3+^, 0.05 Yb^3+^ is dissimilar when excited with the help of a UV lamp. Afterglow images of Na_2_CaGe_2_O_6_ doped with only Tb^3+^ display a rapid decrease in their intensity; thus, co-doping of Yb^3+^ ions was done to optimize the afterglow intensity, as shown in Fig. 20d [443]. Also, the TL glow curves shown in Fig. 20e [443] represent the concentration of charge carriers captured by traps, while the peak in the glow curve shows trap depth. From the photoluminescence curve shown in Fig. 20f [443], it can be observed how variation in the concentration of Tb^3+^ affects the emission spectrum and bi-temporal luminescence. Fig. 20(g–j) [444] represents counterfeiting images of three different portions, viz., butterfly, flower, and leaf, with a pattern of a flower made of Na_2_CaGe_2_O_6_:Pb^2+^ co-doped with Y^3+^, a leaf pattern made with the help of Na_2_CaGe_2_O_6_:Pb^2+^ co-doped with Mn^3+^ and Yb^3+^, and a flower pattern made with the help of Na_2_CaGe_2_O_6_:Pb^2+^ co-doped with Tb^3+^.

#### 6.2.2 PLNPs for latent fingerprinting

**Fig. 21:**
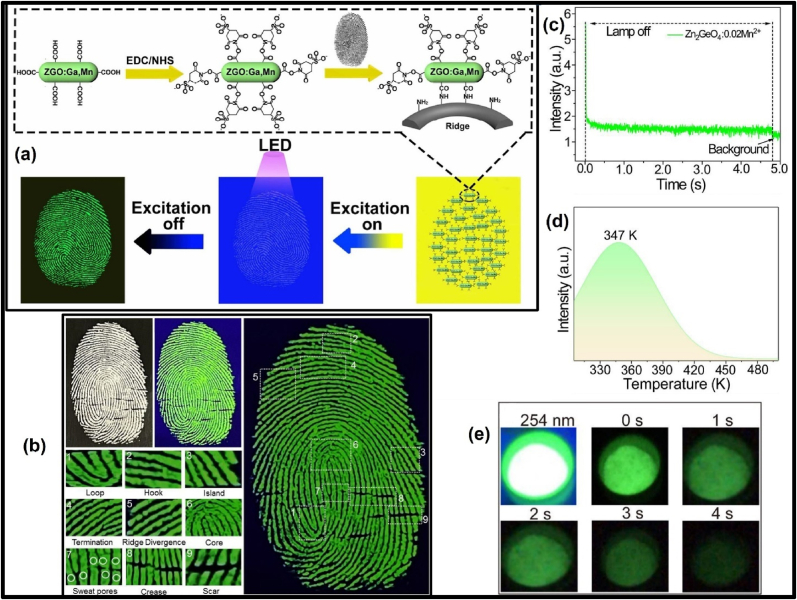
(a) Schematic diagram representing process of identification of fingermarks with the help of Zn_2_GeO_4_:Ga,Mn–COOH nanophosphors Reproduced with permission from Ref. [454]. , copyright 2017, ACS Publications. (b) LPF images obtained from the surface of aluminium foil stained by Zn_2_GeO_4_:Mn^2+^ phosphor under daylight (displaying white color) and after excitation with the help of 254 nm UV lamp (displaying greenish emission). Magnified and enhanced images marked as 1,2,3,4,5,6,7,8,9 display nine kinds of details viz loop, hook, island, termination, ridge divergence, core, sweat pores, crease, and scar respectively (c) intensity vs time graph displaying afterglow curve of Zn_2_GeO_4_:Mn^2+^ observed at 534 nm wavelength. (d) TL glow curve of same nanophosphor indicating concentration of charge carriers in traps. (e) Per Lum images of Zn_2_GeO_4_:Mn^2+^ phosphor at different time intervals after being excited by 254 nm UV light Reproduced with permission from Ref. [457], copyright 2022, Elsevier.

Owing to the overwhelming desire for improved and stronger protection of personal data, highly secure biometrics have seen a boom in the market in recent times. Physical attributes such as fingermarks, speech analysis, facial and retinal scans have been employed to prevent unauthorized persons from accessing facilities or logging on to secure servers [[445], [446], [447], [448]]. Biometrics incorporated into Internet banking systems have been able to ensure safer transactions, secure the collection of data, and provide highly efficient identification at airports [445,447]. Most of the fields related to our day-to-day lives have been made highly secure with the help of such biometric security systems, for example, mobile applications [445,449], forensics, and computer security [448,450,451]. Because of distinctiveness, permanency, inclusiveness, improved collectability, and excellent shelf life, fingermark identification and analysis is by far the most widely used biometric approach [445,447,452]. During the current time, this security technique is used in banks, secure facilities, and other government offices to allow access to sensitive data. Nowadays, computers and cell phones are also equipped with this type of biometric authentication, which has led to more and more daily use of this technique along with the modernization of this technology [447,448]. As PLNPs do not require continuous irradiation because of their longer afterglow, they allow eliminating background noise, which results in an improved SNR ratio. Thus, the use of such materials can result in long-lived and high-resolution fingerprints [453]. Wang et al. [454], synthesized PLNPs Zn_2_GeO_4_:Ga co-doped with Mn via the hydrothermal method in 2017 and used them for constricted identification of background-free latent fingerprints (LFP) as shown in Fig. 21 (a) [454]. They functionalized these nanostructures with the carboxyl group to employ them in the marking of fingerprints that was allowed due to the presence of the amino groups in the protein. Their outcomes showed improved efficiency of Zn_2_GeO_4_:Ga, Mn in effectively minimizing background luminescence, thus allowing supersensitive LFP detection. They could also detect fingermarks at approximately 60 days of age because of the presence of amino acids in the protein molecules contained in the body secretions. Such nanoparticles effectively identified glycoproteins in LFP after they were functionalized with concanavalin A. This technique allowed increased flexibility in fingerprint detection and possessed the potential to reveal the chemical properties of the fingermark, thus rendering it very useful in forensics and clinical applications. For both amorphous and crystalline structures, Tain et al. [455], established in the year 2019 a simple technique for generating a PerLum material with a long afterglow. They synthesized 9-9′-(sulfonyl bis (2,1-phenylene)) bis(9H-carbazole) with the help of intramolecular electronic coupling possessing an ability to emit irradiation with increased afterglow in the presence of cross-linked polymers with only 1 % molar weight of doping. They reported the use of this material with a super-long afterglow for time-gated detection of LPF with the help of just a smartphone and a UV light. They observed that background interference was completely removed by capturing a photograph of fingermarks after switching off the excitation source, thus introducing a new and improved technique for the detection of interference and substrate scattering-free LPF. Zheng et al. [456], established a unique temperature-sensitive approach based on shallow-trap PerLum to accomplish ultra-long detection of fingermarks with improved quality and resolution, which could be detected even after wearing latex gloves. Broadband NIR PerLum was observed on a temperature-sensitive film made from transparent silica gel coated by a Bi_2_Ga_4_O_9_:Eu^3+^ co-doped with Cr^3+^ phosphorescent material, enabling a temperature sensitive detection via a thermo-stimulated luminescence technique. They observed that this material allows highly sensitive thermal detection, which could be detected as a phosphorescent pattern by an EMCCD camera even after a single touch. These PerLum films were observed to have increased temperature sensitivity to tiny thermal variations, thus allowing LPF through rubber gloves within a few seconds. This type of LPF approach promises great potential for thermally sensitive LPF, which does not need continuous excitation for the generation of data for fingermarks. Considering that sensory receptors in humans respond to the green wavelength region, it is very critical to design phosphors with green emission that possess improved efficiency. Xue et al. [457], in the year 2022 employed solid-state reaction method to synthesize Mn^2+^-doped Zn_2_GeO_4_ phosphor, that could generate intense green emission with a highly improved internal quantum efficiency of 98.5 %. Because of the uniqueness of human fingermarks based on the ridge patterns (size approximately equal to 450 μm), how they are related to each other, and the size of sweat pores ranging from 88 to 220 μm, they used these phosphors (size ranging from 5 to 10 μm) for the application of fingerprinting. Fig. 21b [457] shows latent fingerprint images taken on an aluminium foil substrate where the ridge pattern was clearly visible under daylight and a UV lamp emitting photons of 254 nm wavelength. Xue et al., observed a green afterglow of Zn_2_GeO_4_:0.02 Mn^2+^ and detected that its afterglow lasted for 5 s after excitation, as shown in Fig. 21 (c) [457]. They also reported that its emission spectrum at 323 nm exhibits a thin band centering at 534 nm, which could be attributed to the ^4^T_1_ to ^6^A_1_ transition of the activator ion displaying full width at half of the maximum of 49.5 nm. They investigated the temperature-dependent and temperature-sensitive characteristics of the synthesized phosphor by tracking the temperature-sensitive phosphorescent emission intensity and afterglow of this activator ion and discovered that the average peak responsiveness of Zn_2_GeO_4_:0.02Mn^2+^ green-emitting luminescent material is 4.90 % K^−1^ and 0.74 % K^−1^, respectively. For further details about afterglow intensity, duration, and how they depend on electronic traps, they studied TL glow curves as shown in Fig. 21d [457], in which a broadband can be clearly seen being centered at 347 K. They calculated the trap depth of this phosphor to be equal to 0.694 eV, which shows that this phosphor will generate afterglow at room temperature [458]. To observe the afterglow of this nanophosphor, optical photographs were taken at different time intervals (0, 1 s, 2 s, 3 s, and 4 s) after exciting it with UV photons of 254 nm wavelength, as shown in Fig. 21 (e) [457]. Such synthesized luminescent materials exhibit phosphorescence with emission wavelengths lying in the green region of the electromagnetic spectrum, which is confirmed by studying thermoluminescence glow curves for this phosphor. They reported that several multi-mode luminescent outputs, with its help, can be designed for securing data, which includes anti-counterfeiting and LPF with the help of its improved afterglow properties. They reported Zn_2_GeO_4_ doped with Mn^2+^ to possess multifunctional green emissions, which could be used for optical temperature measurement, anti-counterfeiting, LPF, and solid-state lighting applications. The fields of biomedicine and security applications have benefited due to PerLum nanomaterials up to a great extent, as has already been explained in good detail in the review. Apart from these two applications, PerLum also extends to many other fields, which have already been discussed in other reviews [19,50,51,459]. Luminescent markings are mainly used for display and safety signs, which are helpful in preventing or reducing any harm to human life in extreme situations. The advantage of using PerLum-based safety signs is that they can be visible to the naked eye even in the dark and do not need continuous excitation, thus making them environment-friendly, long-lasting, and convenient to use [56,460,461]. Such luminescent materials can also be used as optical data recording devices and sensors by utilizing the trapping of charge carriers. Because of the increased depth of traps for excited electrons or holes, energy can be stored with the help of PerLum nanomaterials for longer durations of time after being excited with photons of a suitable wavelength. This stored energy as trapped charge carriers can be triggered to be released because of thermal, optical, and mechanical force, thus resulting in afterglow emission. As a result, we can employ the PerLum phenomenon for data read and write procedures [[462], [463], [464]]. Photocatalysts are a special type of material that can induce a particular type of chemical change. This type of material makes use of photons generated by the material to induce or speed up reactions that would be problematic, if not impossible, to carry out in the dark. The property of PerLum nanomaterials to generate light for a long enough duration has proved very helpful for their use as photocatalysts [50,465,466]. This phenomenon also has a vast application field for solar cells and solid-state lighting devices [[467], [468], [469]].

## 7 Conclusion

This review presents detailed information on different concepts related to PerLum, such as different mechanisms, synthesis methods, other related fundamental concepts, and some important applications. These nanoparticles possess a unique feature in the sense that they can eliminate autofluorescence because they do not require in-situ activation, thus allowing them to be excellent candidates for bio-imaging-guided drug delivery, photo-thermal, and photodynamic therapy. In previous years, PLNPs of various compositions were synthesized for the improvement of photoluminescence intensity and afterglow duration to get improved results in biomedical and security applications. Variations in different properties, such as synthesis pattern, calcination temperature, type of host, and dopant concentration, lead to the tuning of the PerLum properties and thereby expanded their domain of application. Even after significant improvements have been made in the synthesis method, many areas remain unexplored, such as the tunable synthesis of nanophosphors with novel excitation and emission spectra, improved afterglow duration and luminescence intensity, which will help in discovering new applications via the use of efficient activators and host materials. Because of the variation in the luminescent properties of such nanoparticles in the presence of some particular biological elements, such materials have also been used for biosensing, also. In this review, we also have discussed some recent developments in the fields of multimodal imaging, drug administration, and recent achievements in medical diagnostics and therapeutic areas of PLNPs. Such materials, owing to their peculiar luminescent properties and low background signal, have also been used for applications of anti-counterfeiting and latent fingerprinting.

## Outlook

PLNPs, because of their interesting properties such as longer duration of afterglow, charge carrier trapping, and strong emission intensity, have grabbed the attention of researchers in analytical chemistry and material sciences. Advancements in PerLum for the applications of biosensing drug delivery, photo-thermal and photodynamic therapy, anticounterfeiting, and latent fingerprinting have already been discussed in this review, but there is a lot more scope for further research in such fields to improve their efficiency, intensity, afterglow duration, and tuning of absorption wavelength. Such materials possess a distinct upper hand in reducing autofluorescence and background signal, as they do not need *in situ* excitation and thus are appropriate substances for the above-mentioned applications. For further improving such properties, novel hosts and new activators need to be found. PerLum nanophosphors employed for biosensing have also been observed to suffer from several issues, which include low dispersion and simple aggregation. As a result, further improved biosensor probes and methodologies based on the properties of PerLum are being researched, and future advancements of such materials in this field are still worth exploring. Various issues need to be addressed for PerLum nanoparticles used in bio-imaging applications, such as the use of heavy metal ions, such as REs commonly used, which can cause serious damage to the human body. Thus, it is imperative to develop improved and safe PerLum nanoparticles, which demands further research in this subject.

## Declaration of competing interest

The authors declare that they have no known competing financial interests or personal relationships that could have appeared to influence the work reported in this paper.

## Data Availability

Data will be made available on request.
